# Patterns of Adaptation to Drought in *Quercus robur* Populations in Central European Temperate Forests

**DOI:** 10.1111/gcb.70168

**Published:** 2025-04-03

**Authors:** Tetyana Nosenko, Hilke Schroeder, Ina Zimmer, Franz Buegger, Franziska Orgel, Imke Burau, Prasath Balaji Sivaprakasam Padmanaban, Andrea Ghirardo, Ronja Bracker, Birgit Kersten, Jörg‐Peter Schnitzler

**Affiliations:** ^1^ Helmholtz Zentrum München Research Unit Environmental Simulation Neuherberg Germany; ^2^ Thünen Institute of Forest Genetics Grosshansdorf Germany

**Keywords:** adaptation, carbon isotope ratio, common garden, drought tolerance, intrinsic water use efficiency, postglacial recolonization, *Quercus robur* (pedunculate oak), spring phenology

## Abstract

In order to predict the future of European forests, it is crucial to assess the potential of the dominant perennial species to adapt to rapid climate change. The aim of this study was to reconstruct the pattern of distribution of drought tolerance in *Quercus robur* in the current center of the species' range. The distribution and plasticity of drought‐related traits in German populations of *Q. robur* were assessed and the effects of spring phenology and species demographic history on this distribution were evaluated using a drought stress experiment in a common garden. We show that variation of drought‐related functional traits, including intrinsic water use efficiency (iWUE), leaf osmotic potential (π), and rate of drought‐induced defoliation, is high within *Q. robur* populations. However, frequency of trees with high estimated constitutive drought tolerance increases with decreasing water availability in the regions of population origin, indicating local adaptation to drought. A strong correlation between the distribution of drought‐related traits and spring phenology observed in *Q. robur* suggests that adaptation to water deficit interacts with adaptation to the strong seasonality of the central European climate. The two processes are not influenced by the history of post‐glacial recolonisation of central Europe. The results of this study provide a basis for optimistic prognoses for the future of this species in the center of its current distribution range.

## Graphical abstract

We examined drought tolerance of pedunculate oak from German populations. Our results show that trees from drier regions have greater drought tolerance, suggesting that they actively adapt to their local environment. Moreover, we observed a link between drought tolerance and the timing of spring bud burst, suggesting that adaptation to water stress may be evolving alongside seasonal shifts in tree development—another major consequence of climate change. These adaptations are driven by current environmental conditions, but do not reflect the pattern of post‐glacial recolonization. Our results suggest that pedunculate oak has strong potential to withstand future climate challenges.

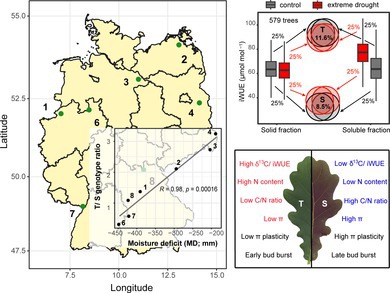

## 1 Introduction

Global warming leads to an increase in the frequency and duration of drought events in Europe. According to the climate projections, many historically humid and sub‐humid regions of central Europe are expected to shift to semi‐arid conditions by the end of this century (Donmez et al. 2023; Tripathy et al. 2023). Reduced water availability threatens the health of established forest ecosystems and, in the long run, can lead to species succession (Thom et al. 2023). There are three possible fates for the perennial species under rapid climate change: persistence through migration, persistence through adaptations to new conditions at current habitats, and extirpation (Aitken et al. 2008). Estimating the potential of the dominant perennial species to adapt to drought is important for predicting and managing the future of European forests.

*Quercus robur* and *Quercus petraea* are the dominant oak species in central Europe. In Germany, they account for more than 9% of forest trees (Blickensdörfer et al. 2024). The two species colonized central Europe during the postglacial period via common migration routes and occupy overlapping ranges (König et al. 2002; Petit et al. 2002). Within these ranges, the two species have different site preferences related to different water requirements (Eaton et al. 2016). *Q. petraea* tends to occupy habitats with lower precipitation and poor soils and has a greater ability to cope with water deficit than *Q. robur* . Thus, it has been shown that in the mixed natural stands in France, *Q. petraea* trees had higher intrinsic water use efficiency (iWUE), a parameter estimated based on carbon isotope ratio in leaf and wood (δ^13^C; i.e., the ratio of stable carbon isotopes ^13^C and ^12^C being fixed by RuBisCo), higher leaf conductance, and a lower vulnerability to drought‐induced xylem embolism than neighboring *Q. robur* trees (Epron and Dreyer 1993; Ramirez‐Valiente et al. 2020; Skelton et al. 2021; Kebert et al. 2022; Le Provost et al. 2022; Rabarijaona et al. 2022). Several scenarios for the future of European oak forests predict the replacement of *Q. robur* by *Q. petraea* through natural migration accelerated by introgressive hybridization, at least at its southern margins (Thuiller et al. 2005; Konatowska et al. 2024).

The present study focuses on variation in drought tolerance within and among *Q. robur* populations in Germany, the current center of the species' range.

Drought tolerance is a complex trait (Pfenninger et al. 2024). It involves root and leaf morphology, vascular tissue anatomy, and the ability to rapidly acclimate to the water deficit through solute accumulation, transpiration regulation, partial defoliation, and other morpho‐physiological and molecular adaptations. This complexity, combined with different water retention strategies among species adapted to different habitats, makes the selection of optimal parameters for quantifying drought tolerance challenging (Kaproth et al. 2023). Several morpho‐physiological traits related to water retention have been used to compare drought tolerance between and within oak species (Epron and Dreyer 1993; Ramirez‐Valiente et al. 2020; Skelton et al. 2021; Kebert et al. 2022; Le Provost et al. 2022; Rabarijaona et al. 2022). These studies often yield controversial results due to the complexity of the trait, differences in experimental design, and the coordinated adaptation of oaks to multiple interacting and alternating environmental stressors. Therefore, in the present study, we first established parameters for estimating drought tolerance in *Q. robur* by comparing the drought‐induced changes in δ^13^C and other phenotypic above‐ground traits, such as stomatal conductance (SC), pre‐dawn leaf water potential (Ψ_PD_), and osmotic potential (π) with its sister species *Q. petraea* . We then use these parameters to assess the patterns of drought tolerance variance in German populations of *Q. robur* .

We address the following questions:

(i) Are there differences in drought tolerance among the *Q. robur* populations from different regions of Germany? (ii) Does local adaptation account for differences in drought tolerance, if any, among *Q. robur* populations? (iii) How does the distribution of traits related to drought tolerance interact with adaptation to seasonal changes in environmental conditions? In particular, we assess the correlation between drought tolerance and variation in bud burst (BB) time across populations. (iv) Are differences in drought tolerance among populations related to the history of the postglacial oak recolonization of central Europe?

Our answers to these questions will help to assess the potential of *Q. robur* to adapt to future environmental conditions in the center of its range and to develop a forest management strategy.

## 2 Materials and Methods

### 2.1 Plant Material

**FIGURE 1:**
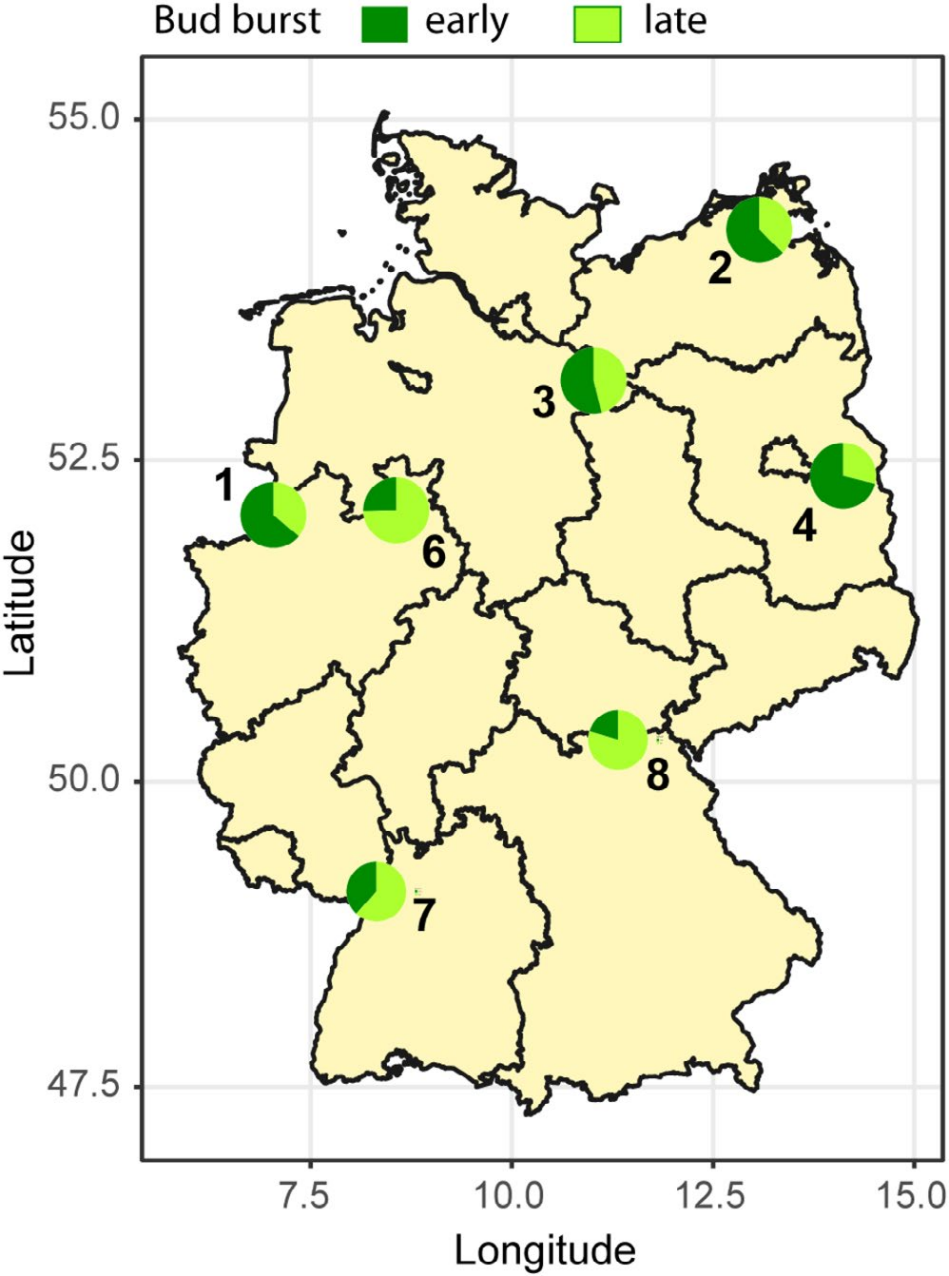
Map of oak populations included in this study with bud burst time distribution. Pie charts show the proportion of the early (dark green) and late (light green) bud burst (BB) trees in the seven oak populations in a common garden at Grosshansdorf, Germany (519 *Q. robur* and 60 *Q. petraea* trees). The pie chart position on the map corresponds to the regions of population origin. Geographical coordinates of the populations are in Table S1. IDs are assigned to the populations as follows: P1—West German lowland bay, P2—Northeast German lowlands, P3—Northwest German lowlands, P4—Southeast German basins and hills, P6—Central low mountain ranges and Harz, P7—Upper Rhine valley, P8—Ore mountains, Thuringian Forest. Map lines delineate study areas and do not necessarily depict accepted national boundaries. The trees were divided into two cohorts based on the BB observations in the years 2020 and 2021 (Table S1). Maximum difference in the bud burst time between the common garden trees was 28 days (21 day in population 2); the 2‐years average difference was 1 week.

Seven hundred and eight two‐year‐old *Quercus robur* (L.) and *Q. petraea* (Matt.) Liebl. seedlings from seven German populations grown from seeds in a common substrate were obtained as root‐naked plants from the tree nursery Schrader Pflanzen Handelsgesellschaft GmbH & Co. (Kölln‐Reisiek, Germany) in March 2019 (Figure 1, Table S1). They were potted in 3 L pots in a standard soil mix (Supporting information) and distributed across the outdoor common garden area of the Thünen Institute for Forest Genetics, Grosshansdorf, Germany using a randomized block design with 10 replicate blocks (3 × 3 trees each) per population, and maintained under mesic conditions with supplemental irrigation. In the winter of 2021, before the experiment, the trees were repotted in 15 L pots. To differentiate between *Q. robur* , *Q. petraea* , and their hybrids, all common garden trees were subjected to the molecular analyses using six nuclear markers and a protocol described in Schroeder and Kersten (2023). All hybrids (11) and damaged trees were excluded from this study. Bud burst (BB) was monitored annually; the basal stem diameter and height were measured in 2022 before the experiment (Table S2).

### 2.2 Design of the Common Garden Drought‐Stress Experiment

The drought stress experiment was conducted in June–July 2022 with 519 *Q. robur* genotypes from seven populations. In addition, 60 *Q. petraea* trees from a habitat with limited water availability (population P2) were included in the experiment as a reference for drought tolerance. Trees were divided into two experimental cohorts based on the average BB index in 2020–2021 (Supporting information and Table S2). The BB index corresponds to the stage of leaf development observed for each individual tree on the day with the highest among‐trees variation for this trait. Trees with an earlier BB time were assigned a higher BB index. To account for the variance in leaf age due to different BB times, the experiment was conducted with the two cohorts, early and late BB trees, with a 1‐week interval (20.06–18.07 and 27.06–24.07.2022, respectively). Sixty‐five mini‐blocks, each containing up to nine trees of similar size, were randomly distributed throughout an open‐sided tunnel (9–11 blocks per population; Figure S1). After saturating the soil water content (SWC), irrigation was stopped until SWC dropped below 12%. SWC was measured in each pot every other day using a Moisture Meter HH2 with soil moisture sensor ThetaProbe type ML2x (Delta‐T Devices, Cambridge, UK). For all 579 trees, leaves from the previous year's shoots from the upper sun‐exposed part of the canopy were sampled twice around midday (11 am—3 pm): on the first day of the experiment (control samples) and after a period of drought when SWC had dropped below 12% (extreme drought). Samples were snap frozen in liquid nitrogen and stored at 80°C for further assessment of nitrogen (N) and carbon (C) concentrations, δ^13^C, and leaf osmotic potential (π). Air temperature and humidity were measured twice daily at nine positions in the tunnel using a HygroPalm 3 (Rotronic AG, Bassersdorf, Switzerland). At the end of the experiment, the drought‐induced defoliation was visually estimated for each tree as the percentage of dry or discolored leaves. To control for drought stress, Ψ_PD_ and SC were measured twice a week in a subset of trees of average height (175–193 cm; 38 early‐ and 44 late BB trees) in the center of the experimental area (sets 1 and 2; Figure S1), including 4 days of the post‐drought recovery period. Ψ_PD_ was measured in the first‐year green shoots collected from the top of the tree canopy at 3–5 am CET using the SAPS II Plant Water Status Console model 3115 (Soil moisture Equipment Corp., Santa Barbara, USA). After measurements, leaves from these shoots were snap frozen in liquid nitrogen for further analysis of N, C, δ^13^C, and π. SC was measured in the sun‐exposed leaves at midday using a Leaf Porometer SC‐1 (Decagon Devices, Pullman, USA).

### 2.3 Leaf Osmotic Potential Measurements

To estimate leaf osmotic potential (π), 10 mg of dry leaf tissue powder was resuspended in 750 μL of 10 mM potassium phosphate pH 7.0 (KPi). The osmolarity of this solution (50 μL) was measured using a Röbling type 13 freezing‐point osmometer (Röbling Messtechnik GmbH, Berlin). The osmolarity of the buffer was subtracted from the leaf sample measurements, and π was calculated using the Van't Hoff equation as described in Callister et al. (2006):

$$\pi =-\frac{\left(\frac{n}{V}\right)\mathrm{RT}\;\mathrm{DW}}{\left(\frac{m}{V}\right)\left(\mathrm{FW}-\mathrm{DW}\right)}$$

where (n/V) is osmolarity of the leaf extract (mOsm kg^−1^); *R* is the universal gas constant; *T* is the absolute temperature (°K) calculated based on the buffer molarity; (m/V) is the ratio of the leaf dry weight to the extract volume, and (FW—DW) is the difference between fresh and dry weight.

Genotype‐specific π plasticity was calculated as the difference between extreme drought and control values.

### 2.4 Elemental and Stable Isotope Analyses

Analysis of ^13^C‐stable isotope and elemental analysis of C and N content were performed in the solid and soluble fractions of leaf tissue using an Isotope Ratio Mass Spectrometer (IRMS; delta V Advantage, Thermo Fisher, Dreieich, Germany) coupled to an Elemental Analyzer Euro EA (Eurovector, Milano, Italy), using established procedures (Simon et al. 2013). Details can be found in Supporting information. The data are available from Nosenko and Schnitzler (2025).

The δ^13^C values of the samples were expressed relative to the international VPDB standard:

$$\delta^{13}C\;\left(‰\text{VPDB}\right)=\left(\left(R_{\text{sample}-}R_{\text{VPDB}}\right)/R_{\text{VPDB}}\right)\;1000$$

where *R*_sample_ is the ^13^C/^12^C ratio of the sample and *R*_VPDB_ = 0.0111802 (Werner and Brand 2001). Technical error and between‐branch variance of δ^13^C were estimated as described in Supporting information. The plasticity of δ^13^C was estimated for each genotype as the difference between δ^13^C under the extreme drought and control conditions.

### 2.5 Calculation of Intrinsic Water Use Efficiency

Intrinsic water use efficiency (iWUE) values were calculated from δ^13^C in the soluble and solid fractions of leaf extracts using the R package isocalcR (Mathias and Hudiburg 2022). Mean temperatures for the period of leaf development (21.04–11.07.2022) were calculated from the daily temperature information of the German Weather Service (DWD) station 1975 (https://opendata.dwd.de/). Atmospheric carbon isotope ratios (δ^13^C_atm_) were obtained from the atmospheric CO_2_ and δ^13^C_atm_ database (Belmecheri and Lavergne 2020).

### 2.6 Determination of Leaf Abscisic Acid Content

Abscisic acid (ABA) content in leaves of selected genotypes with high and low iWUE was determined using an ultra‐performance liquid chromatography system Ultimate 3000RS UPLC (Thermo Fisher, Bremen, Germany) coupled with ultra‐high resolution tandem quadrupole/time‐of‐flight mass spectrometry (Impact II QqToF mass spectrometer; Bruker, Bremen, Germany) according to a modified protocol of Zhou et al. (2023) and quantified using Metaboscape 4.0 (Bruker). The detailed protocol can be found in Supporting information.

### 2.7 Identification of *Quercus robur* Chloroplast DNA Haplotypes

To assess the effect of oak demographic history on the iWUE distribution in *Q. robur* in Germany, chloroplast DNA (cpDNA) haplotype data were generated for the 519 *Q. robur* genotypes using the KASP technique and the cpDNA locus *trnDT* (1600 bp; eight marker‐SNPs and one indel (Degen et al. 2021)). This analysis allows for the discrimination between five *Q. robur* haplotype groups, including haplotypes 1 (origin—Italy), 4, 5, and 7 (Balkans), and haplotype group 10/11/12 (merged Iberian Peninsula lineages; Petit et al. 2002). KASP data were generated from total genomic DNA at LGC Genomics. Total DNA was extracted from oak leaves according to Bruegmann et al. (2022). The data are available from Nosenko and Schnitzler (2025).

### 2.8 Climate Information

Climate information for the period of 1991–2020 and climate‐derived soil parameters for 1991–2010 were obtained from the DWD Open Data Portal (https://opendata.dwd.de/; Table S1). DWD stations in the vicinity of the population origin regions (< 20 km) were identified using the population geocoordinates and the R package RDWD v. 1.8.0 (Boessenkool 2023). Moisture deficit (MD) was calculated as the difference between mean annual evaporation over grass and loamy sand (VGLS) and mean annual precipitation. The aridity index (Ia) was calculated as the ratio of mean annual precipitation to potential evaporation VPGB computed by the agrometeorological model AMBAV (Braden 2013). Actual soil parameters, including soil water holding capacity (FC), soil water available to plants (PAW), effective rooting depth (ER), and soil air content (AC), were extracted from the Soil Atlas of Germany database (https://bodenatlas.bgr.de/). The month of the growing season with the lowest precipitation level (April) and the month with the most variable precipitation across Germany (June) were inferred based on the monthly precipitation data for the period of 1991–2020 at 5583 DWD stations. Minimum daily temperature information for the period of leaf development in the common garden in 2022 was obtained from the DWD station 1975.

### 2.9 Statistical Analyses

Statistical analyses were performed using R 4.3.2 (R Core Team 2023). The ranges of within‐ and among‐population variance in δ^13^C and δ^13^C plasticity were calculated separately for control and extreme drought conditions using the R package dplyr (Wickham et al. 2023). All physiological (Ψ_PD_, π, SC, SWC), morphological (stem height and basal diameter, BB index, drought‐induced defoliation), metabolic (leaf δ^13^C, C, N, C/N ratio, iWUE), and genetic (cpDNA haplotype) parameters, as well as population‐specific climate and soil variables were analyzed by fitting a generalized linear model using the gml function in the lme4 R package (Bates et al. 2015). The best‐fitting model was selected based on the Akaike information criterion (AIC) using the AUCmodavg R package (Mazerolle 2023). As climate and soil variables were often collinear, only models with a single climate/soil predictor were included to avoid overfitting. Exploratory plots of correlations between variables were constructed using the R packages corrplot (Wei and Simko 2021) and ggplot2 (Wickham 2009). For all correlation analyses, linear regression was performed in R using the lm function. The effects of condition, population, and BB on δ^13^C, π, and δ^13^C and π plasticity were estimated using two‐way ANOVA with the aov R function; the post hoc analysis was conducted using the TukeyHSD test. Following the method of Joswig et al. (Joswig et al. 2022), we used median trait values per population to test for trait changes across environmental gradients. The Wilcoxon signed‐rank test was used to assess differences in various parameters between species, populations, and conditions.

## 3 Results

### 3.1 Carbon Isotope Ratio Is a Reliable Parameter for Assessing Drought Tolerance

**FIGURE 2:**
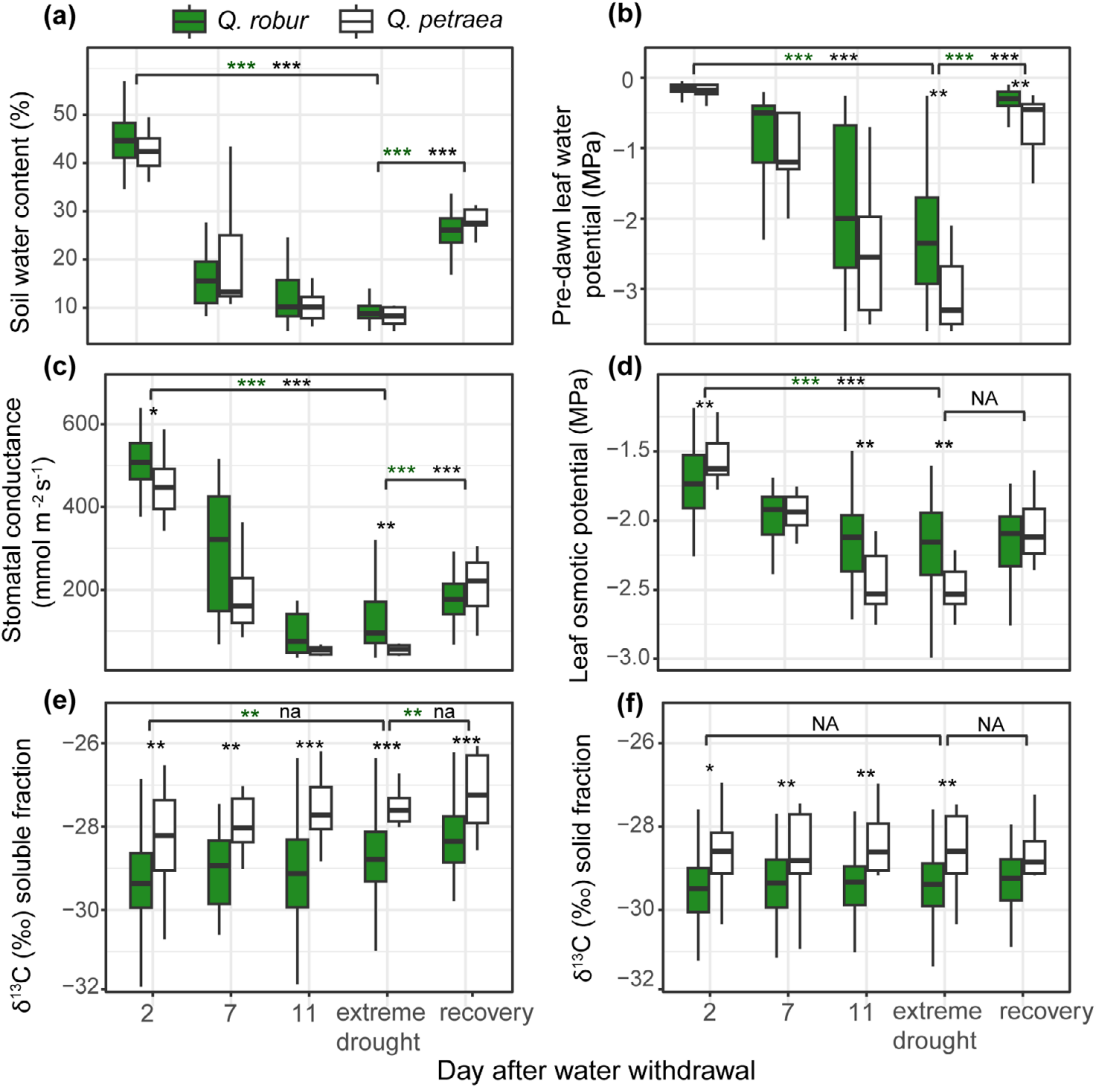
Dynamics of drought stress responses in *Quercus robur* and *Q. petraea* trees. Dynamics of (a) soil water content (SWC) and associated changes in (b) pre‐dawn leaf water potential, (c) stomatal conductance, (d) leaf osmotic potential, and (e, f) carbon isotope ratio (δ^13^C) in leaf soluble and solid fractions are shown for 72 *Q. robur* and 10 *Q. petraea* trees from sets 1 and 2 during the first 11 days after the water withdrawal (before any tree included in the experiment has been re‐watered), under extreme drought, and after 4 days of recovery. All control measurements were performed at day 2. “Extreme drought” corresponds to the day when SWC fell below 12%; it varied from 11 to 21 days after water withdrawal depending on the plant. The thick line in each box shows the median. The lower and upper limits of the box (hinges) correspond to the first and third quartiles. The upper whiskers extend to the maximum value but no further than 1.5 of the interquartile range (IQR) from the hinge. The lower whiskers extend to the minimum value but not further than 1.5 IQR from the hinge. Stars above boxes indicate significant between‐species difference (Wilcoxon test *p*‐value * < 0.05; ** < 0.01; *** < 0.001). Stars above brackets indicate significant differences between conditions in *Q. robur* (in green) and *Q. petraea* (in white).

To establish parameters and thresholds for estimating drought tolerance in *Q. robur* , we compared the dynamics of physiological and metabolic changes induced by changing SWC in *Q. robur* from the seven regions of Germany with *Q. petraea* from a region characterized by relatively low precipitation (P2, Northeastern German Lowlands; 82 trees from sets 1 and 2; Figure 1 and Figure S1). Ψ_PD_, SC, and π correlated with SWC (Wilcoxon test *p* < 0.001; Figure S2). The dynamics of Ψ_PD_, SC, and π were similar in the two species (Figure 2). All three decreased in response to the decrease in SWC (*p* < 2.2e^−16^ for both species). After rewatering, Ψ_PD_ and SC increased rapidly (*p* < 2.2e^−16^), while π did not change significantly. Under extreme drought, Ψ_PD_, SC, and π were lower in *Q. petraea* from P2 compared to *Q. robur* from different populations (*p* < 0.05), which is consistent with the higher drought tolerance of *Q. petraea* trees from the low‐precipitation region. However, the differences between species for these traits were unstable. In contrast, δ^13^C proved to be a rather stable parameter. Both δ^13^C and, in the soluble fraction, the δ^13^C dynamics differed between species (Figure 2e,f and Figure S2d). In *Q. petraea* , δ^13^C did not change significantly throughout the experiment, but remained higher than in *Q. robur* in both soluble and solid fractions (*p* < 0.05), suggesting drought adaptation in *Q. petraea* . *Q. robur* , however, showed greater δ^13^C plasticity compared to *Q. petraea* : the decrease in SWC triggered a gradual increase in δ^13^C in the soluble fraction, which continued even during the plant recovery phase (*p* < 0.01). These differences in δ^13^C and δ^13^C dynamics could have implied different strategies for coping with drought between the two species. However, the comparison between *Q. petraea* and *Q. robur* from a single mixed stand (P2; 83 trees) showed a difference in δ^13^C only under extreme drought and only in the soluble fraction (*p* < 0.05; Figure S3), suggesting that the constitutive δ^13^C level and δ^13^C plasticity in both species may be determined by the environmental conditions in the region of population origin.

Estimation of the variation between technical replicates showed that the technical δ^13^C error was negligible (Table S3). The δ^13^C variation within *Q. robur* genotypes (i.e., between branches) was significantly lower than between genotypes and, in the soluble leaf fraction, between conditions. Other traits, including C and N content and the C/N ratio, showed neither a significant difference between species nor any significant change in response to drought (Figure S4).

In summary, the interspecies comparison showed that δ^13^C, especially in the soluble fraction, is the most stable and reliable parameter for assessing drought tolerance in *Q. robur* . Hereafter, δ^13^C refers to measurements in the soluble fraction unless otherwise stated. δ^13^C was measured in 579 common garden oaks under normal irrigation and extreme drought conditions. Although π was found to be less stable, we included this parameter in our analyses as an additional potential criterion for estimating drought tolerance in *Q. robur* .

### 3.2 Distributions of Leaf Carbon Isotope Ratio and Leaf Osmotic Potential Depend on the Population and the Bud Burst Time

**FIGURE 3:**
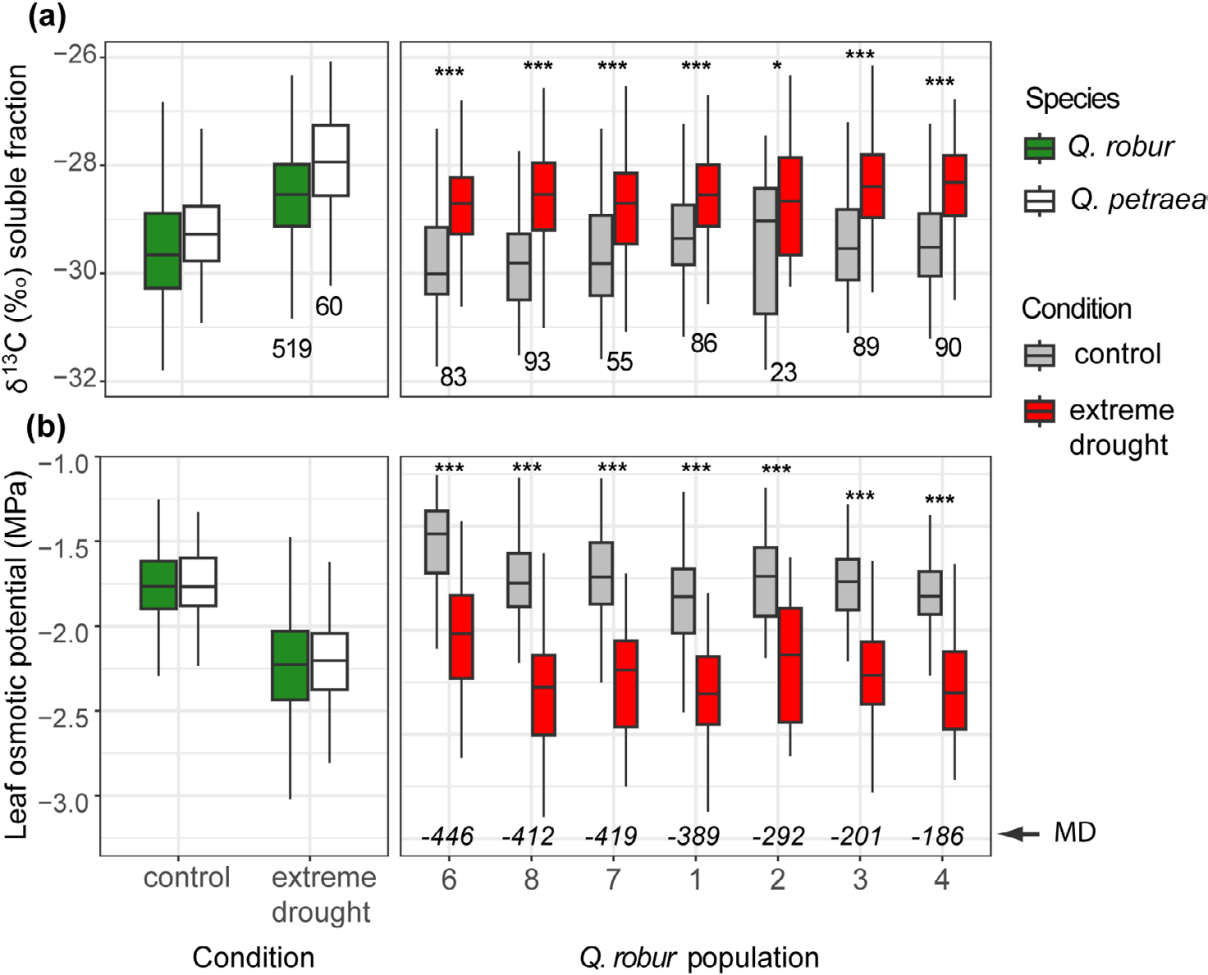
Variation of carbon isotope ratio and leaf osmotic potential between oak species and populations. (a) Carbon isotope ratio (δ^13^C) and (b) leaf osmotic potential (π) are compared between 519 *Q. robur* trees from seven German populations (green) and 60 *Q. petraea* trees from population P2 (Northeast German lowlands; white) and between *Q. robur* populations under normal irrigation conditions (gray) and drought (red). Population order corresponds to moisture deficit at the regions of population origin (MD). The number of trees of each species and in each population is shown below boxes in (a). MD is shown for each population above the *x*‐axis in (b). Stars indicate significant difference between conditions as follows: Wilcoxon test *p*‐value * < 0.05, *** < 0.001. The box‐plot statistics is as in Figure 2.

Analyses of the distribution of δ^13^C, π, and their plasticity (i.e., drought‐induced changes) revealed high variance in these traits within and among *Q. robur* populations, with variation within populations being higher than variation among populations (Figure 3 and Table S4). A significant proportion of *Q. robur* genotypes had δ^13^C values above the median δ^13^C values of *Q. petraea* from the region with relatively high MD, both under normal irrigation and extreme drought conditions (36% and 23%, respectively). Over 49% of *Q. robur* trees had π below the median π of *Q. petraea* under both conditions. Such a distribution suggests a high adaptive potential in *Q. robur* in Germany.

We found significant effects of condition, population, and BB time on both δ^13^C and π distributions in *Q. robur* (*p* < 0.0001; Table 1). The population effect was partly due to the difference between genotypes from the low‐ and high‐MD regions. In particular, in P6, a population from the low‐MD region, δ^13^C was lower compared to P3 and P4 from the high‐MD regions (Tukey HSD test difference 0.35‰–0.41‰, *p* < 0.01) and π was higher compared to all other populations (0.15–0.31 MPa, *p* < 0.0001). P1 was outstanding among populations from the low‐MD regions. It had higher δ^13^C (0.29‰–0.41‰, *p* < 0.05) and lower π (0.11–0.24 MPa, *p* < 0.001) than P6, P7, and P8. In addition, δ^13^C plasticity was lower in P1compared to P6, P8, and P3 (0.35‰–0.39‰, *p* < 0.05).

**TABLE 1:** Effects of population, condition, and bud burst time on trait variance in *Quercus robur* .

	δ^13^C (‰)^a^ *F* (*p*)	δ^13^C plasticity^a^ (‰) *F* (*p*)	π (MPa) *F* (*p*)	π plasticity (MPa) *F* (*p*)
Population	6.12 (2.6e^−06^)	3.20 (0.004)	28.24 (< 2.2e^−16^)	na
Condition	350.87 (< 2.2e^−16^)	—	1072.1 (< 2.2e^−16^)	—
BB index	161.59 (< 2.2e^−16^)	34.76 (6.7e^−09^)	151.1 (< 2.2e^−16^)	na
Population‐BB index	6.28 (1.7e^−06^)	na	7.48 (7.3e^−08^)	na

^a^ Data are given for the soluble leaf tissue fraction; F—ANOVA F‐statistics.

The effect of BB time was due to the correlation of this trait with δ^13^C, δ^13^C plasticity, and π (*p* < 2.2e^−16^; Figure S5). In the early BB trees, δ^13^C was higher, whereas π was lower than in late BB trees under both conditions (difference 0.63‰ and 0.16 MPa, respectively; *p* < 0.0001; Figures S6 and S7). In the high‐MD populations, δ^13^C plasticity was lower in early BB trees (0.35‰; *p* < 0.0001). The distribution of daily minimum temperatures at the Grosshansdorf experimental site during the leaf development period in May–June 2022 makes priming by low temperature an unlikely cause of the differences between the two groups (Figure S8).

The statistical effect of the interaction between population and BB time terms on δ^13^C and π was due to the uneven distribution of early and late BB trees across populations (Figure 1). For example, early BB trees make up over 64% of P1 but only 25% of P6, which has significantly lower δ^13^C and higher π than P1.

Another trait that correlated positively with δ^13^C and negatively with π (*p* = 8.2e^−07^ and 9.4e^−06^, respectively) was drought‐induced defoliation, indicating a higher percentage of leaf loss in the presumably drought‐tolerant genotypes. Tree height and diameter had no effect on δ^13^C and δ^13^C plasticity but showed a negative correlation with π (*p* = 1.9e^−05^ and 1.5e^−06^, respectively). The correlation of π with tree size, together with its sensitivity to SWC fluctuations, makes this parameter less reliable for determining drought tolerance in oak.

The factors that may have contributed to the population effect on the trait variation are local adaptation and species demographic history, including species migration and regional differences in forest management in Germany.

### 3.3 Climatic Clines in Trait Distributions Reflect Synergism of Drought Adaptation and Phenological Shift

**FIGURE 4:**
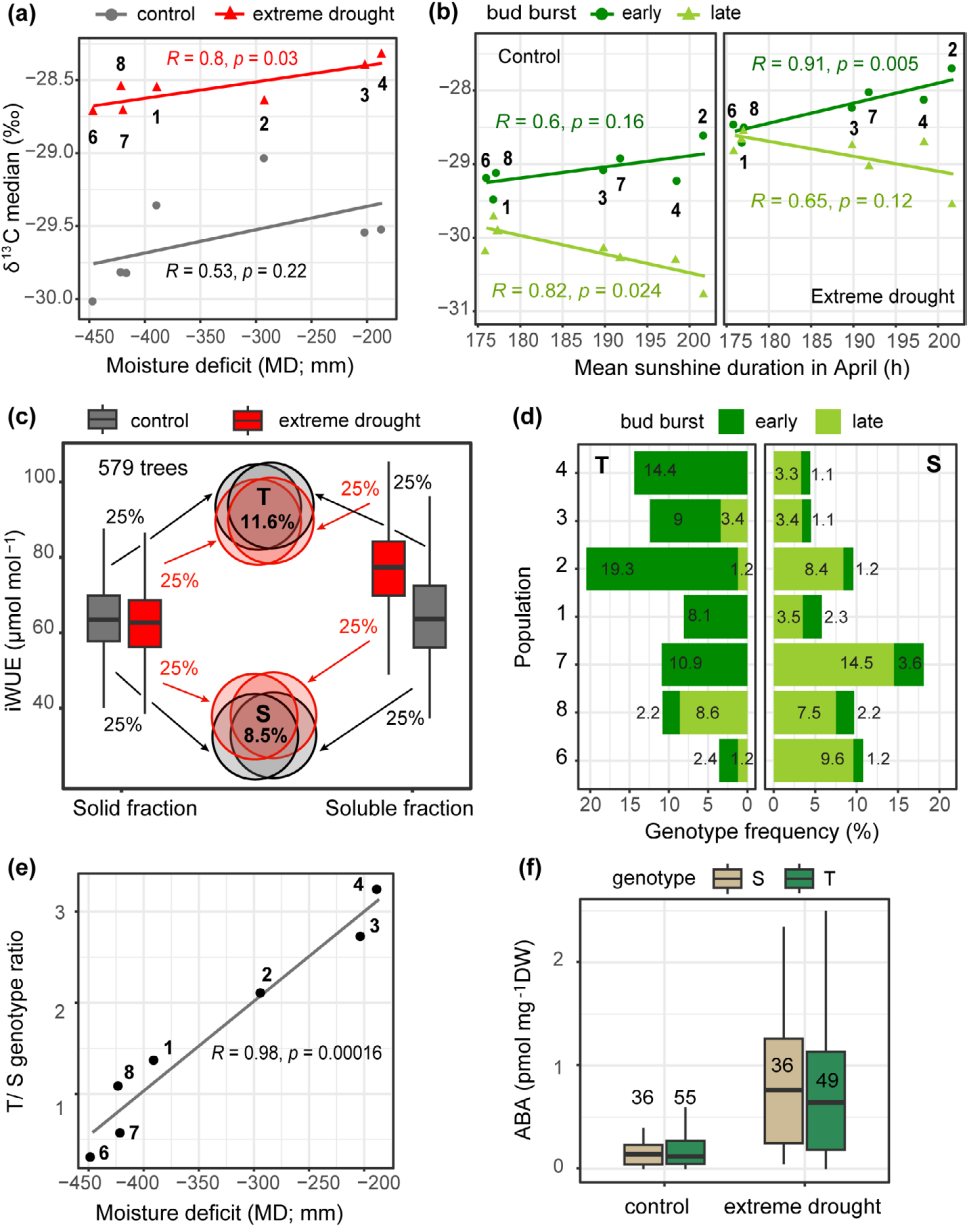
Local adaptation to water availability in oak in Germany. (a) Correlation between the median of carbon isotope ratio (δ^13^C) in the soluble fraction of *Quercus robur* leaves under normal irrigation and extreme drought (SWC < 12%) conditions and mean annual moisture deficit (MD) in the seven regions of population origin. (b) Correlation between leaf δ^13^C in early and late bud burst (BB) *Q. robur* trees under normal irrigation and extreme drought conditions and mean multiannual sunshine duration in April. (c) Selection of trees with high and low intrinsic water use efficiency (iWUE; T and S for drought tolerant and susceptible, respectively). T and S trees were selected from the total pool of 579 common garden trees based on four types of measurements: IWUE in solid and soluble leaf tissue fractions under control (gray) and extreme drought (red) conditions. Genotypes that occur in the upper and the lower iWUE quartiles in all measurement groups were selected as T and S, respectively. The statistical method for the boxplot is as in Figure 2. (d) Across‐population distribution of T and S oak ( *Q. robur* and *Q. petraea* ) trees. The dark green and light green bar segments and the numbers at the top of each segment show the percentage of T and S trees with early and late BB, respectively. Population P2 includes both species. Population order corresponds to MD (low to high). (e) Correlation between the T and S ratio and MD in the population origin regions. Dots and triangles are labeled with corresponding population IDs. Population IDs are as in Figure 1. (f) Concentration of abscisic acid (ABA; pmol mg^−1^ dry weight) in leaves of T and S genotypes under normal irrigation and extreme drought conditions.

In common garden experiments, correlations between traits and functionally relevant environmental variables are considered to be an indication of local adaptation (Hancock et al. 2011). In the present study, we observed a significant correlation between the population median of δ^13^C under drought and seven climate and soil variables (*p* < 0.05; Figure 4a and Figure S9). These correlations were negative for the mean annual precipitation in the regions of population origin, precipitation during the growing season, precipitation during the driest month of the growing season (i.e., April), Ia, and FC, whereas correlations with MD and AC, a parameter opposite to FC, were positive. All seven variables are relevant to water availability and strongly correlate with each other (Figure S10).

The genotype‐specific BB time proved to be a constant parameter, showing a strong correlation between three consecutive years (*p* < 2.2e^−16^; Figure S5). Despite high within‐population variance, the BB index in *Q. robur* in 2020–2022 correlated negatively with the seven climate and soil variables relevant to water availability (*p* < 1e^−14^). In contrast to δ^13^C, this trait also correlated with the mean annual number of hot days and annual and monthly sunshine hours in the regions of population origin. The latter correlation was strongest in April, the month of BB initiation in most of the trees (*p* < 2.2e^−16^). Interestingly, median δ^13^C formed opposite clines in early and late BB trees when populations were distributed by both sunshine duration and mean annual number of hot days (Figure 4b and Figure S11). There was no statistically significant interaction between either the BB index or δ^13^C and mean annual and monthly air temperatures in the regions of population origin.

The climatic clines in the leaf δ^13^C and BB time distributions, as well as the association between the two traits, reflect coordinated adaptation of *Q. robur* to multiple interacting environmental stressors. The δ^13^C distribution suggests increased drought tolerance in oak populations from regions with lower precipitation, Ia, and FC and higher MD and AC, which is consistent with the local adaptation to water deficit in these populations. To further test this prediction, we used iWUE—a drought tolerance parameter calculated from δ^13^C.

### 3.4 Distribution of Trees With Extremely High and Low iWUE Supports Local Adaptation to Water Availability

To estimate the relative frequencies of drought‐tolerant and drought‐susceptible trees within populations, we selected individuals with iWUE in the upper and lower quartiles of the iWUE data pooled for all 579 *Q. robur* and *Q. petraea* trees (T and S, respectively; Figure 4c). A strong correlation between the iWUE in the soluble and solid fractions and, within each fraction, between drought and control (*p* < 1e^−84^; Figures S12 and S13) suggests that using all four types of measurements may help to exclude outliers resulting from either technical error or inter‐branch variance. Therefore, only trees that occurred in all four high and low iWUE groups were selected as T and S, respectively. The resulting subsets of T and S trees comprised 25% and 5% of the total *Q. petraea* and 10% and 8.9% of the *Q. robur* trees, respectively. The frequencies of T and S varied between populations. The frequency of T tended to increase and the frequency of S tended to decrease with increasing MD in the regions of population origin (Figure 4d). Thus, in *Q. robur*, the frequency of T was highest and the frequency of S was lowest in P4, characterized by the highest MD (14.4% and 4.4%, respectively), while P6, at the opposite end of the MD distribution, had the lowest frequency of T (3.6%). The T to S ratio correlated negatively with precipitation, Ia, and FC and positively with MD and AC (*p* < 0.01; Figure 4e).

Consistent with the correlation between δ^13^C and BB index, over 82% of T trees were characterized by early BB, while the majority of S trees (79.8%) had a delayed BB (Figure 4d). In addition, T had significantly higher N content and lower C/N ratio in both solid and soluble leaf tissue fractions and lower π compared to S under both normal irrigation and extreme drought conditions (Figure S14). Despite the correlation between the drought‐induced defoliation and iWUE observed in the 519 *Q. robur* trees, we found no significant difference for this trait between T and S (Figure S14f). The concentration of ABA, a hormone involved in the regulation of drought response, did not differ between T and S and showed no correlation with δ^13^C and π (Figure 4f and Figure S15). This is consistent with the fact that the differences observed between T and S were not condition dependent.

Both the correlation of δ^13^C with environmental variables and the trend observed in the population‐wide distribution of trees with extreme iWUE are consistent with the effect of local adaptation to water availability, but do not exclude the possibility of a demographic effect.

### 3.5 Chloroplast DNA Haplotype Differentiation has no Effect on iWUE and Bud Burst Time Variation

**FIGURE 5:**
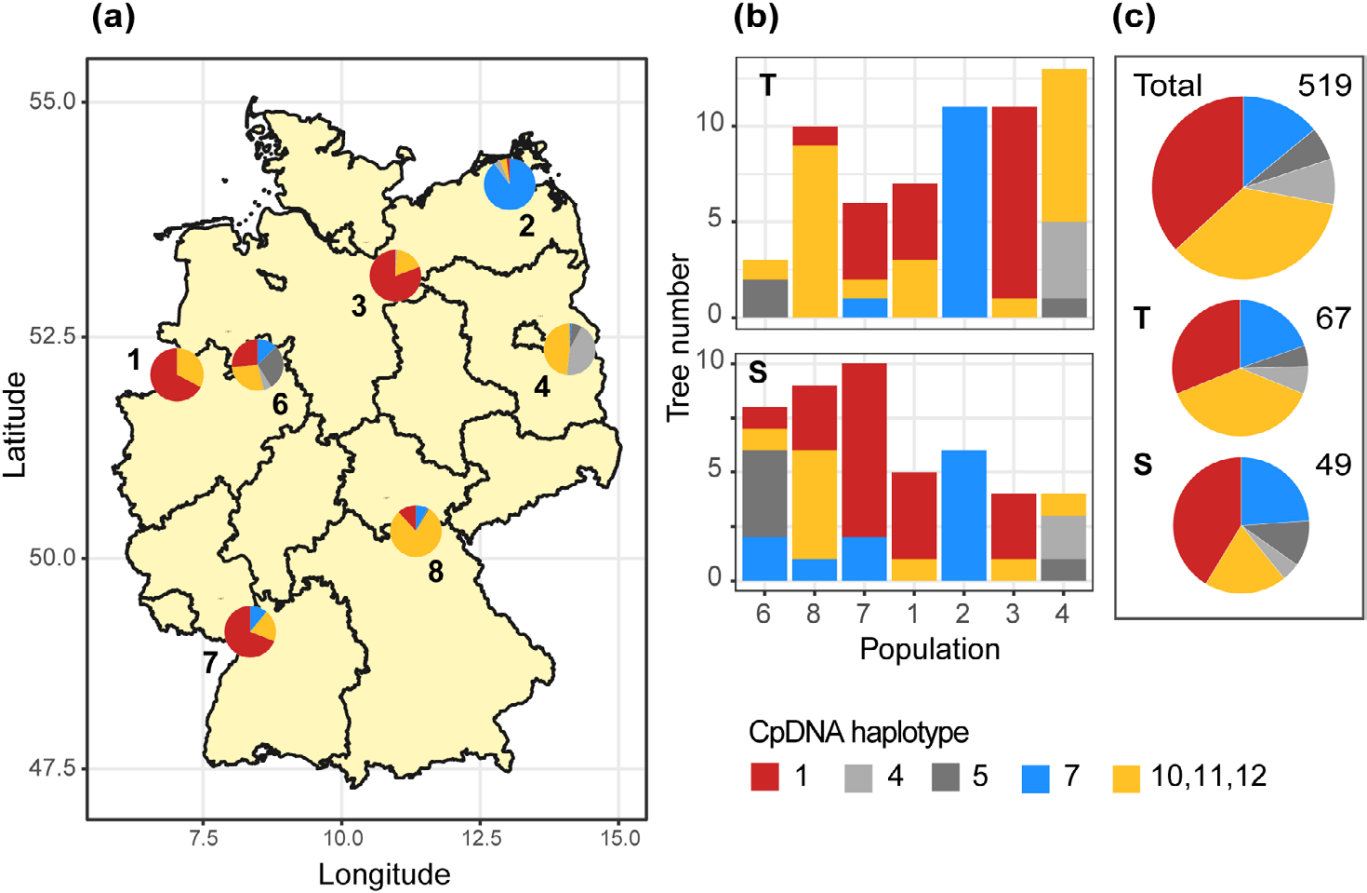
Effect of the postglacial recolonization history on the distribution of drought tolerance in *Quercus robur* in seven German populations. (a) Map of *Q. robur* cpDNA haplotypes. Pie charts show the *Q. robur* cpDNA haplotype composition of the populations. (b) CpDNA haplotype composition of the subsets of *Q. robur* trees with extremely high and low intrinsic water use efficiency (iWUE; T and S, respectively) in different populations. Population order corresponds to moisture deficit (MD; low to high). Population IDs are as in Figure 1. (c) Pie charts showing the total cpDNA haplotype composition of all *Q. robur* trees included in the drought stress experiment and subsets of trees with extreme iWUE values (T and S). CpDNA haplotype IDs and colors are as in Petit et al. (2002): Red—haplotype 1 originating from the Italian peninsula; orange—a group including Iberian Peninsula haplotypes 10, 11, and 12; light gray—haplotype 4, Balkans; dark gray—haplotype 5, Balkans; blue—haplotype 7, Balkans. Our analysis does not discriminate between three Iberian haplotypes 10, 11, and 12. Map lines delineate study areas and do not necessarily depict accepted national boundaries.

The maternally inherited cpDNA makes it possible to trace the history of postglacial recolonization of central Europe by *Q. robur* via three major migration routes (König et al. 2002; Petit et al. 2002). To test whether the pattern of δ^13^C/iWUE distribution observed in the German *Q. robur* populations was due to differences in their cpDNA haplotype composition, we identified the cpDNA haplotypes of 515 out of the 519 *Q*. *robur* trees (Table S1). All *Q. robur* populations were polytypic (i.e., containing two or more haplotypes; Figure 5a). The haplotype composition differed between populations. The haplotype frequencies and distributions across Germany largely followed the general remigration pattern described in König et al. (2002), although the approximately equal representation of five haplotypes in P6 may be due to historical human impact. All cpDNA haplotypes showed high δ^13^C variance; each haplotype included both T and S trees (Figure 5 and Figure S16a,b). The cpDNA haplotype composition of subsets of trees with extreme iWUE values was almost identical to the Germany‐wide haplotype composition. Furthermore, cpDNA haplotypes showed no association with BB time or drought‐induced defoliation that could not be explained by climate in the regions of population origin (Figure S16c,d).

Taken together, these results suggest that adaptation to drought in *Q. robur* of different cpDNA lineages occurred locally within each population. Postglacial migration has no apparent effect on this process.

## 4 Discussion

### 4.1 Local Adaptation to Water Availability in *Quercus robur*

Variation in response to stress is determined by genetic adaptation (i.e., heritable genomic changes) and phenotypic plasticity (Rowland et al. 2023). Although plasticity is traditionally defined as phenotypic adjustments to environmental fluctuations that do not require genetic changes, recent studies have shown that the amplitude of these adjustments is to some extent under genetic control (Bhaskara et al. 2022). An association between the inter‐population differences in functional traits or their plasticity observed in common gardens and water availability in the regions of population origin is considered to be a strong indication of local adaptation to drought (Hancock et al. 2011; Skelton et al. 2021).

In the present study, we observed a high variation of leaf δ^13^C, a standard estimator of iWUE, within German populations of *Q. robur*. The δ^13^C variance among populations was much lower than within populations. Such a distribution is typical for long‐lived perennial species and can be attributed to large population sizes, high gene flow, and adaptation to habitats characterized by complex interactions and alternation of multiple abiotic and biotic constraints (Niinemets 2010; Chamaillard et al. 2011; Cavender‐Bares 2019; Rabarijaona et al. 2022; Hamrick et al. 1992). Despite the low variance among populations, we observed significant differences in δ^13^C between several populations originating from German habitats with contrasting MD and soil FC. Population median values of δ^13^C in *Q. robur* correlated with climate and soil variables relevant to water availability in the regions of population origin. These climatic clines in the δ^13^C distribution were statistically significant only under the extreme drought. In the common garden study, which included Europe‐wide provenances of *Q. petraea* , Rabarijaona et al. (2022) observed an association between the δ^13^C plasticity (but not δ^13^C itself) in mature trees and soil characteristics at provenance sites. In contrast, we found no correlation between the δ^13^C plasticity in *Q. robur* and any climate or soil variable, although several populations differed for this trait. Therefore, plasticity alone does not explain the fact that, despite a significant correlation between the control and drought δ^13^C values, the climatic δ^13^C clines were only observed under extreme drought in *Q. robur* . This clinal pattern results from a combination of inter‐population differences in δ^13^C plasticity and constitutive δ^13^C. Recently, Morillas et al. (2024) reported higher constitutive δ^13^C values in seedlings of the evergreen oak *Q. suber* from xeric Mediterranean habitats compared to trees from mesic habitats. This observation suggests that constitutively high δ^13^C is a genetic adaptation to drought, which is consistent with studies showing that in both *Q. robur* and *Q. petraea* δ^13^C/iWUE levels are determined by a small number of genes with a large effect (Brendel et al. 2008; Le Provost et al. 2022). Furthermore, these genes showed contrasting expression patterns between *Q. petraea* genotypes with high and low iWUE, indicating different molecular strategies to cope with drought (Le Provost et al. 2022). Genetic determination suggests that relative iWUE values should remain constant among trees of the same age growing under the same conditions. However, evaluating differences in genetically determined iWUE in mature trees is difficult due to the additional factors that contribute to iWUE variation, including greater variation between branches of the same canopy, differences in competitive success between trees, and variation in microhabitat (le Roux et al. 2001; Rabarijaona et al. 2022). For example, greater root depth, which is another trait contributing to drought tolerance (Abrams 1990), and/or a better access to groundwater may result in a lower iWUE for individual trees.

In our study, we observed an increased frequency of *Q. robur* trees with constitutively high δ^13^C/iWUE in populations from habitats characterized by relatively low water availability. The population ratios of high to low iWUE trees also showed a strong correlation with MD and other climate and soil parameters related to water availability. Analyses of the distribution of extreme iWUE trees support local adaptation to drought in *Q. robur* in Germany, but also show that all German populations contain both high and low iWUE trees. These observations have important implications for the development of future forest management strategies.

### 4.2 Interaction of Drought‐Related Traits With the Time of Bud Burst

Another trait that showed a clinal distribution along environmental gradients in *Q. robur* was BB time. It correlated with the same climate and soil variables as δ^13^C, but also with the mean annual number of hot days and mean sunshine duration, especially in April, the month of BB initiation in *Q. robur* in Germany. Of the two related climatic factors, sunshine duration and water availability, the former is more likely to influence BB time. *Q. robur* is known to be sensitive to photoperiod (Basler and Korner 2014). Variations in sunshine duration due to differences in cloud cover between regions may have the same effect on BB time as latitudinal shifts in day length. The BB time varied up to 21–28 days within *Q. robur* populations. Individual trees showed a consistent phenological ranking over 3 years, suggesting genetic differentiation for this trait (Wesołowski and Rowiński 2006; Malyshev et al. 2022). In the central European climates characterized by pronounced seasonality, maintaining a high BB time variance increases population fitness by enabling some individuals to escape spring frost damage and others to avoid herbivorous insects and benefit from an extended growing season (Crawley and Akhteruzzaman 1988). We also observed a significant correlation between BB time and δ^13^C, which could potentially be attributed to the association between sunshine duration and water availability. However, this association does not explain the relative distribution of these traits within populations: significantly higher δ^13^C and lower π and, in some populations, δ^13^C plasticity in early BB trees compared to late BB trees. Furthermore, δ^13^C in early and late BB trees formed opposite clines when populations were distributed by sunshine duration or annual number of hot days. No such pattern was observed in relation to precipitation and other variables relevant to water availability.

In natural habitats, two non‐exclusive causes for higher leaf δ^13^C and osmolyte concentrations in early BB trees would be possible: (1) genetically determined adaptations that allow early BB trees to survive spring episodes of low temperature and (2) acclimation to drought or cold at an early stage of leaf development (in this case, before leaf unfolding in late BB trees). The design of our experiment and the temperature distribution at the experimental site during BB make the second cause unlikely. Both δ^13^C and π are related to drought tolerance. Therefore, the observed pattern implies a complex interaction between drought tolerance and adaptation to the short growing season in *Q. robur*. In plants, both drought and cold affect the water balance and carbon metabolism and therefore trigger similar physiological responses regulated by overlapping molecular pathways. Acclimation to both stresses involves stomatal closure, solute accumulation, and long‐term reallocation of carbohydrates from growth to defense (Kim et al. 2024). A genetically determined tolerance to low temperature (cause 1) may be advantageous for a plant to withstand drought episodes. To our knowledge, the association between iWUE and BB time has not been reported previously. However, using a combined genome‐environment and genome‐phenome association study, Leroy et al. (2020) found both precipitation‐associated SNPs and leaf unfolding‐associated SNPs within two important genes controlling stomatal regulation, the ROPGEF1 and APK1b, in *Q. robur* and *Q. petraea* . As δ^13^C reflects the efficiency of stomatal regulation (Cernusak et al. 2013), these genes may provide a molecular link between the two traits.

Coordinated adaptation to multiple co‐occurring environmental constraints contributes to the genetic and phenotypic variation in oak populations, and thus presents a challenge for designing common garden experiments (Gimeno et al. 2009). This may have contributed to the lack of association found between climatic gradients and δ^13^C/iWUE in studies that included Europe‐wide oak populations (Jucker et al. 2017; Torres‐Ruiz et al. 2019).

### 4.3 Effect of Postglacial Recolonization on the Distribution of Drought‐Related Traits

The history of the postglacial recolonization has shaped the cytoplasmic genetic diversity of oak species in central Europe (König et al. 2002; Petit et al. 2002). According to chloroplast genome analyses, three major genetically distinct *Q. robur* lineages originating from the Italian, Iberian, and Balkan peninsulas entered central Europe via different recolonization routes. Accordingly, the cpDNA haplotype composition of the oak population varies between regions and may potentially influence the distribution of phenotypic traits among populations. Because of this concern, phenotypic and genomic studies of oak species are often limited to a single cpDNA lineage (Le Provost et al. 2022; Rabarijaona et al. 2022).

In the present study, five *Q. robur* cpDNA haplotypes were identified. All populations were polytypic, consisting of 2–5 haplotypes. We found no difference between cpDNA haplotypes in any of the phenotypic traits analyzed, including leaf δ^13^C and its plasticity, N and C content, C/N ratio, π, BB time, and drought‐induced defoliation. The haplotype compositions of high and low iWUE genotype subsets did not differ from each other or from the German‐wide haplotype distribution. We conclude that the cpDNA haplotype distribution has no effect on the clinal distributions of δ^13^C and BB time observed in *Q. robur*. Both traits are controlled by nuclear genes (Scotti‐Saintagne et al. 2004; Singh et al. 2018; Le Provost et al. 2022). Although the original postglacial differentiation of the chloroplast genome still persists, over 6000 years of gene flow, recombination, and local adaptation have probably erased this differentiation from the nuclear genomes in *Q. robur*. This is consistent with the conclusion of Kremer et al. (2002) derived from the analyses of cytoplasmic and nuclear controlled variation in *Q. petraea* populations.

### 4.4 Phenotypic Characteristics of Trees With High and Low Intrinsic Water Use Efficiency

**FIGURE 6:**
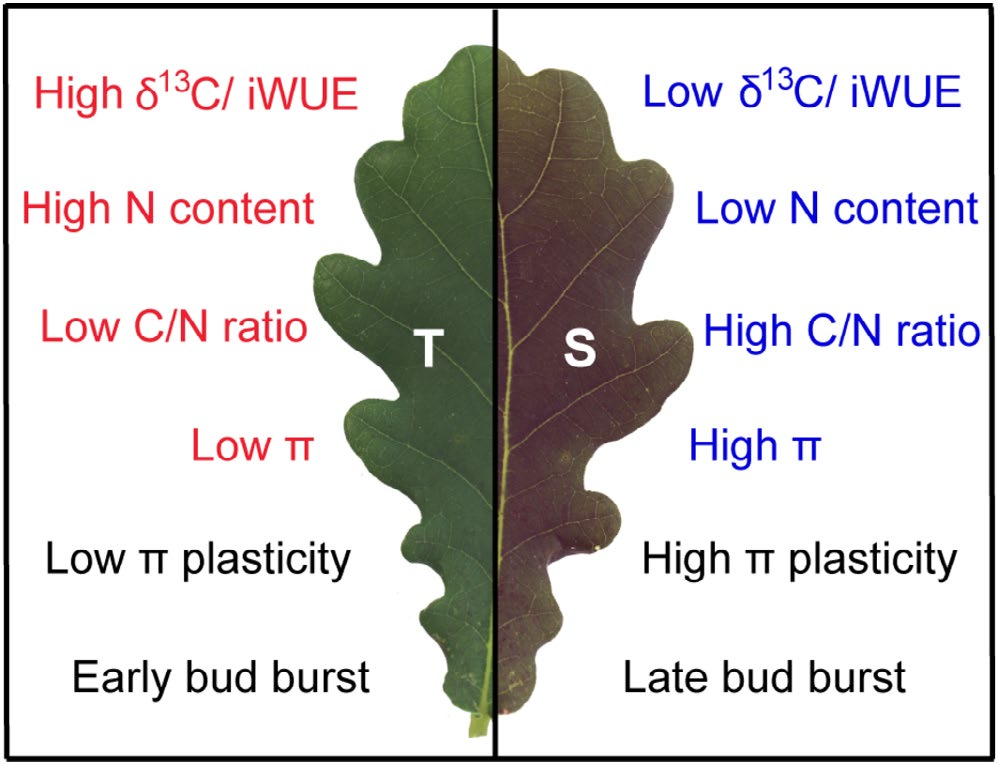
Schematic summary of phenotypic differences between *Quercus robur* trees with constitutively high and low intrinsic water use efficiency (iWUE). T and S refer to oak trees with high and low iWUE, respectively. General traits of drought‐tolerant plants are shown in red; traits typical for drought‐susceptible plants are shown in blue.

Comparison of high and low iWUE trees showed that in addition to δ^13^C, the trait directly related to iWUE, the two groups differed in other phenotypic traits (Figure 6). In particular, N content was higher, while C/N ratio was lower in the high iWUE trees compared to the low iWUE trees regardless of irrigation status. Higher leaf N content is associated with increased photosynthetic capacity. It is particularly beneficial in plants from arid environments as it allows carbon assimilation to be maintained at reduced SC (Wright et al. 2003). High iWUE trees also had significantly lower π under normal irrigation and drought conditions, whereas π plasticity was higher in the low iWUE trees, indicating different strategies for coping with drought. Finally, most of the high iWUE trees had an early BB time, while most of the low iWUE trees had a late BB time. Regardless of whether the association between BB time and iWUE is causal or non‐causal, this trait should be considered in genome‐wide association studies aimed at determining the genetic basis of drought tolerance in *Q. robur* .

## 5 Conclusion

High variation in both constitutive levels and plasticity of drought‐related traits observed within German populations of *Q. robur* reflects high potential for drought adaptation in this species. Although drought remains random and precipitation is still greater than the rate of evaporation (expressed as negative MD) in the study region, our results show that drought adaptation is an ongoing process in *Q. robur* in the temperate forests. Furthermore, the strong association between the drought‐related traits analyzed and spring phenology observed in *Q. robur* may suggest an evolutionary synergism between drought adaptation and phenological shift, another consequence of global climate change. Taken together, the high phenotypic variation within populations and ongoing local adaptation provide a basis for optimistic prognoses for the future of this species in the center of its current distribution range.

## Author Contributions

**Tetyana Nosenko:** conceptualization, data curation, formal analysis, investigation, methodology, writing – original draft, writing – review and editing. **Hilke Schroeder:** conceptualization, data curation, writing – review and editing. **Ina Zimmer:** investigation, methodology, writing – review and editing. **Franz Buegger:** investigation, writing – review and editing. **Franziska Orgel:** investigation, writing – review and editing. **Imke Burau:** investigation, writing – review and editing. **Prasath Balaji Sivaprakasam Padmanaban:** formal analysis, investigation, writing – review and editing. **Andrea Ghirardo:** investigation, methodology, writing – review and editing. **Ronja Bracker:** investigation, writing – review and editing. **Birgit Kersten:** conceptualization, funding acquisition, project administration, writing – review and editing. **Jörg‐Peter Schnitzler:** conceptualization, funding acquisition, project administration, writing – original draft, writing – review and editing.

## Conflicts of Interest

The authors declare no conflicts of interest.

## Supporting information


Table S1.

Table S2.

Supporting information.

## Data Availability

The data that support the findings of this study are openly available in Open Science Framework at https://doi.org/10.17605/OSF.IO/AMHGQ. Climate and soil data were obtained from the DWD Open Data Portal at https://opendata.dwd.de/ and Soil Atlas of Germany database at https://bodenatlas.bgr.de/, respectively.
