# Supplementary material for: Patterns of Adaptation to Drought in Quercus robur Populations in Central European Temperate Forests
Source: Glob Chang Biol. 2025 Apr 3;31(4):e70168. doi: 10.1111/gcb.70168 (PMC11966185; doi:10.1111/gcb.70168)
Supplement: Supplementary file 3 — Supporting information. [file GCB-31-e70168-s001.pdf]

Supporting Information

Article title: Patterns of local adaptation to drought in *Quercus robur* populations in central European temperate forests.

Authors: Tetyana Nosenko, Hilke Schroeder, Ina Zimmer, Franz Buegger, Franziska Orgel, Imke Burau, Prasath Balaji Sivaprakasam Padmanaban, Andrea Ghirardo, Ronja Bracker, Birgit Kersten, Jörg-Peter Schnitzler

The following Supporting Information is available for this article:

Methods S1. Standard container substrate

Methods S2. Tree grouping based on bud burst time

Methods S3. Protocol for elemental and stable isotopes analyses

Methods S4. Estimation of the carbon isotope ratio ($\delta^{13}C$) technical error and inter-branch variation

Methods S5. Protocol for extraction and LC-MS analysis of abscisic acid (ABA) content

Table S1. Oak populations included in this study with climate and soil characteristics of the population origin regions (*as a separate xlsx file*)

Table S2. Phenotypic and cpDNA haplotype information for the common garden trees of *Quercus robur* and *Q. petraea* (*as a separate tab-separated txt file*)

Table S3. Technical error and biological variation in leaf carbon isotope ratio ($\delta^{13}C$) measurements

Table S4. Within- and among-population ranges of carbon isotope ratio ($\delta^{13}C$), leaf osmotic potential (π), and $\delta^{13}C$ and π plasticity in *Quercus robur*

Figure S1. Common garden drought stress experiment with *Quercus robur* and *Q. petraea*

Figure S2. Correlation between soil water content (SWC) and leaf physiological and metabolic parameters in oak

Figure S4. Drought response in *Quercus robur* and *Q. petraea* trees from population P2 (Northeast German lowlands)

Figure S4. Changes in the N and C contents in leaves of *Quercus robur* and *Q. petraea* during the drought stress experiment

Figure S5. Correlations between the drought response traits in *Quercus robur* from seven German populations

Figure S6. Effects of location at the experimental site and bud burst time on the distribution of carbon isotope ratio (δ^{13}) in oak trees from seven German populations

Figure S7. Effects of location at the experimental site and bud burst time on leaf osmotic potential in oak trees from seven German populations

Figure S8. Air temperature at the common garden site at Großhansdorf, Germany during leaf development in oak trees in 2022

Figure S9. Correlation between carbon isotope ratio ($\delta^{13}C$) in the soluble fraction of *Quercus robur* leaves and climatic and soil parameters

Figure S10. Correlation between climate and soil variables in the regions of oak population origin in Germany

Figure S11. Climatic clines in the distribution of carbon isotope ratio in *Quercus robur* trees with early and late bud burst

Figure S12. Correlation between intrinsic water use efficiency (iWUE) in soluble and solid fractions of oak leaves

Figure S13. Correlation between intrinsic water use efficiency (iWUE) in oak leaves under normal irrigation and extreme drought conditions

Figure S14. Trait variation between selected *Quercus robur* genotypes with high and low intrinsic water use efficiency (iWUE)

Figure S15. Correlation between the concentration of abscisic acid (ABA) and intrinsic water efficiency (iWUE) and leaf osmotic potential (π) in selected drought-tolerant and susceptible oak genotypes

Figure S16. Trait variation in the *Quercus robur* cpDNA haplotypes in Germany

Methods S1. Standard container substrate

per m^3:
60 % white peat
20 % mixed peat
15 % wood fiber
5 % cocopeat
75 kg wet clay
1.2 kg PG mix 14-16-18 (Yara, Brunsbüttel, Germany)
50 g micronutrient slow-release fertilizer Radigen
500 ml soil surfactant
pH: target value 5.8
Structure: coarse

Methods S2. Tree grouping based on the bud burst time

Leaf development (first flush) of each tree in the common garden was monitored from the appearance of at least one swollen bud in at least one tree until all leaves were fully developed in all trees in the years 2019-22. We followed the classification of leaf developmental stages (integers from 1 to 5) described in Bertic *et al*. (2021). Trees at intermediate leaf development stages were assigned fractional numbers (*e.g.*, 1.5, 2.5, etc.). To account for the variation in bud burst (BB) time, the common garden oak trees were divided into two groups: early BB trees and late BB trees, - based on the average of BB indices in 2020 and 2021, which correspond to the leaf developmental stage at week four from the beginning of BB. Week four was chosen because it has the greatest variation in leaf development between trees (from stage 0 to stage 4). Trees with an average BB index above 2.5 were considered as "early"; trees with an average BB index of 2.5 or below were considered as "late". BB data from 2019 were not included because tree shipping and re-potting could affect the BB time in some trees. Data from 2022 were not included because the organization of the drought stress experiment, including a randomized distribution of plants across the trial area, took place in the spring of 2022, prior to the BB start. The maximum difference in the full leaf development time (stage 5) between trees was 28 days (21 day for population P2). The average difference between the early and late BB groups of trees was one week. Based on these results, the drought stress experiment with the early and late BB trees was conducted with seven-day interval on 20.06 - 18.07 and 27.06 - 24.07.2022, respectively.

Method S3. Protocol for elemental and stable isotopes analyses

Analysis of ^{13}C-stable isotope ($d^{13}C$) and elemental analysis of C and N content were performed in the soluble and solid leaf tissue fractions. Leaf samples were dried at 65°C for 72 h and ground using a ball mill. 15 mg of dry leaf powder was resuspended in 500 µl 10 mM KPi, incubated for 5 min at 60°C in an ultrasonic bath, and centrifuged for 5 min at 14000 rpm. The supernatant was transferred in a fresh tube, and the procedure was repeated with 250 µl KPi. For the soluble fraction, the supernatants were combined, and 2 x 100 µl were transferred into tin capsules (IVA Analysentechnik, Meerbusch, Germany). For the solid fraction, the pellet was resuspended in 750 µl KPi, and 2 x 100 µl were

transferred in the tin capsules. All samples were dried overnight at 65°C and analyzed using an Isotope Ratio Mass Spectrometer (IRMS) (delta V Advantage, Thermo Fisher, Dreieich, Germany) coupled to an Elemental Analyzer Euro EA (Eurovector, Milano, Italy Analysis of ^{13}C-stable isotope ($d^{13}C$) and elemental analysis of C and N content were performed in the soluble and solid leaf tissue fractions. Leaf samples were dried at 65°C for 72 h and ground using a ball mill. 15 mg of dry leaf powder was resuspended in 500 µl 10 mM KPi, incubated for 5 min at 60°C in an ultrasonic bath, and centrifuged for 5 min at 14000 rpm. The supernatant was transferred in a fresh tube, and the procedure was repeated with 250 µl KPi. For the soluble fraction, the supernatants were combined, and 2 x 100 µl were transferred into tin capsules (IVA Analysentechnik, Meerbusch, Germany). For the solid fraction, the pellet was resuspended in 750 µl KPi, and 2 x 100 µl were transferred in the tin capsules. All samples were dried overnight at 65°C and analyzed using an Isotope Ratio Mass Spectrometer (IRMS) (delta V Advantage, Thermo Fisher, Dreieich, Germany) coupled to an Elemental Analyzer Euro EA (Eurovector, Milano, Italy). Acetanilide (Acros Organics, Geel, Belgium; approximately 0.45 mg) was used as a laboratory standard in each sequence at intervals (after 16 sample measurements). A series of the laboratory standard weights was measured at the beginning of each sequence to determine the isotope linearity of the system. All standard measurements were also used for the calibration and quantification of C and N contents. The laboratory standard was calibrated against the international isotopic standards (International Atomic Energy Agency IAEA, Vienna). Each sequence ended with a final ^{13}C correction using international (IAEA 600, USGS 40, and IAEA C3) and laboratory isotopic standards covering the range of isotopic values of the leaf samples and their C content. Each sample was analyzed in two technical replicates to verify the homogeneity of the sample material.

Method S4. Estimation of the carbon isotope ratio ($\delta^{13}C$) technical error and inter-branch variation

We estimated the contribution of the $\delta^{13}C$ technical error and inter-branch variation (i.e., biological variation between different branches of the same tree) to the within-population variance of this parameter. To estimate the technical error, the laboratory preparation and IRMS measurements were repeated twice for 22 samples. Inter-branch $\delta^{13}C$ variance was estimated based on the difference between $\delta^{13}C$ in samples collected from the first year and the previous year upper crown shoots of trees from sets 1 and 2 under both control and drought conditions (164 samples from 82 trees). The result shows that the variation between the IRMS measurements of technical replicates and different laboratory preparations was negligibly small (SD 0.3% of the mean $\delta^{13}C$). The SD of the $\delta^{13}C$ between the first and previous year branches of the same tree was about 1% of the mean $\delta^{13}C$, which is significantly lower than the SD of $\delta^{13}C$ between genotypes and, in the soluble leaf fraction, between conditions estimated in the same set of trees (Table S3). As the data for the main study were derived from the samples collected from the previous year's shoots, the actual inter-branch variation within the main dataset is expected to be lower than our estimate.

Methods S5. Protocol for extraction and analysis of abscisic acid (ABA)

The abscisic acid (ABA) content in oak leaves was determined according to a modified protocol of Zhou *et al*. (R. *et al.*, 2023). Fifty mg of freeze-dried leaf powder was mixed with 1 mL of extraction solution (acetone/H_2O/acetic acid 80:19:1 v:v:v), ultrasonicated for 5 min, and centrifuged at 5,000 *rpm* for 5 min. The solvent was evaporated in a SpeedVac system for 2 h; the supernatant then was frozen and completely dried by lyophilization at -70°C. For ABA analysis, the residue was resuspended in 100 µL of sample solution (acetonitrile/H_2O/acetic acid; 15:85:0.05 v:v:v), centrifuged at 5,000 rpm for 5 min. The supernatant was then transferred to sample vials. Targeted analysis of ABA (monoisotopic mass 264.13616) was performed using an ultra-performance liquid chromatography (UPLC) system coupled with ultra-high resolution (UHR) tandem quadrupole/time-of-flight (QqToF) mass spectrometry (MS). The instrumentation comprises an Ultimate 3000RS UPLC system (Thermo Fisher, Bremen, Germany), a Bruker Impact II QqToF mass spectrometer, and an Apollo II electrospray ionisation (ESI) source (Bruker Daltonic, Bremen, Germany). Reversed phase-high performance liquid chromatography (RP-HPLC) was performed by injecting 5 µL of sample on a C_{18} column (Acquity BEH, 150 mm × 2.1 mm, 1.7 µm; Waters, Eschborn, Germany). Chromatographic separation was performed using H_2O with formic acid (99.9%; v/v) as aqueous solvent and acetonitrile with formic acid (99.9%; v/v) as the organic solvent, as described in detail elsewhere (Bertic *et al.*, 2021). The MS was operated in negative (-) ionization mode with the following parameters: nebulizer pressure 2.0 bar, dry gas flow 10.0 L/min, dry gas temperature 220°C, capillary voltage 3000 V, endplate offset 500 V, mass range 50–800 m/z, acquisition rate 2 Hz, and rolling averaging of two spectra. Instrument tuning and mass calibration were performed using ESI-L Low Concentration Tuning Mix (Agilent) and 1M sodium formate solution (LiChropur, Merck, Germany), respectively. ABA was quantified by measuring the $[M-H]^-$ ion, extracting the corresponding peak area at a retention time (RT) of 14.85 min using the Metaboscape 4.0 software (Bruker). The concentration of ABA was calculated based on an external dilution series using an authentic ABA standard (Sigma-Aldrich, Taufkirchen, Germany) dissolved in sample solution in the range of 0.5-1.5 ng μL^{-1}.

Table S3. Technical error and biological variation in leaf carbon isotope ratio ($\delta^{13}C$) measurements.

	**$\delta^{13}C$ standard deviation** [Percent of the mean $\delta^{13}C$]	
	**Soluble fraction**	**Solid fraction**
**Laboratory preparation and IRMS measurements***	0.089‰ [0.30%]	0.089‰ [0.30%]
**IRMS measurements**	0.055‰ [0.19%]	0.050‰ [0.17%]
**Inter-branch control****	0.347‰ [1.18%]	0.280‰ [0.95%]
**Inter-branch drought****	0.324‰ [1.14%]	0.279‰ [0.94%]
**Between drought and control****	0.734‰ [2.54%]	0.289‰ [0.98%]
**Between genotypes, control****	1.00‰ [3.4%]	0.800‰ [2.7%]
**Between genotypes, drought****	1.01‰ [3.55%]	0.913‰ [3.1%]

*Based on 44 samples from 22 trees; **based on 164 samples from 82 trees

Table S4. Within- and among-population ranges of carbon isotope ratio ($\delta^{13}C$), leaf osmotic potential (π), and $\delta^{13}C$ and π plasticity in *Quercus robur*.

	**Within-population range**		**Among-population range**	
	**$\delta^{13}C$ (‰)**	**π (MPa)**	**$\delta^{13}C$ (‰)**	**π (MPa)**
**control**	3.9 – 4.9	0.8 – 1.9	0.4	0.3
**drought**	4.1 – 5.9	1.0 – 1.4	0.5	0.3
**plasticity**	3.2 – 4.4	1.0 – 2.2	0.3	0.2

Figure S1. Common garden drought stress experiment with *Quercus robur* and *Q. petraea*. (**a**) Oak trees inside the open side tunnel. (**b**) Map of Tree distribution. Each outlined cell in the plot area corresponds to one tree. Letters and numbers indicate columns and rows containing blocks of nine trees. Colors are assigned to different populations as follows: (1) West German lowland bay - gray, (2) Northeast German lowlands - yellow, (3) Northwest German lowlands - orange, (4) Southeast German basins and hills - green, (6) Central low mountain ranges and Harz - blue, (7) Upper Rhine valley - red, (8) Ore mountains, Thuringian forest - purple. In total, the experiment included 519 *Q. robur* trees from the seven populations and 60 *Q. petraea* trees from population P2. The white line separates the early and late bud burst (BB) groups of trees. Trees were divided into the two groups based on BB observations in the years 2020 and 2021 (Table S2). The two-year average difference in BB time between the two groups was one week. The drought stress experiment with the early and late BB trees was conducted at seven-day interval on 20.06 - 18.07 and 27.06 - 24.07.2022, respectively. The shaded areas in the center of each group, outlined with solid red lines, indicate the intensive study sets of trees (sets 1 and 2). In addition to the parameters estimated for all trees included in the drought stress experiment, sets 1 and 2 were also used to monitor physiological (stomatal conductance, leaf osmotic potential, and pre-dawn leaf water potential) and metabolic (N and C content, N/C ratio, and carbon isotope ratio) changes in oak trees during the experiment and estimating the between-branch variation of the metabolic measurements. (**c**) Daytime ranges of temperature

and humidity in the tunnel during the experiment. (**d**) Distribution of the mean temperature of the hottest day of the experiment (30.06.2022) over the experimental area. Each outlined cell corresponds to a block of nine trees

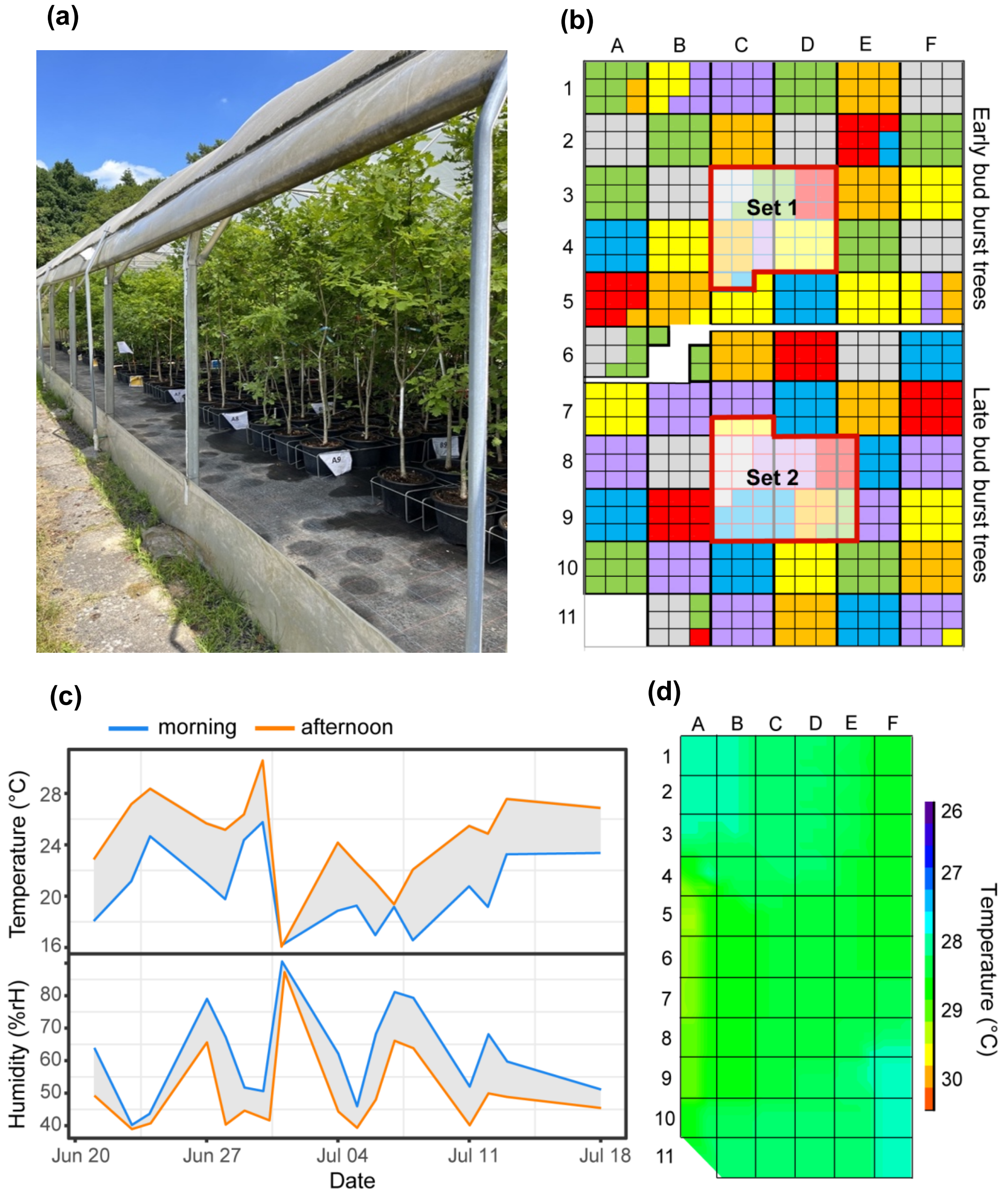

Figure S2. Correlation between soil water content (SWC) and leaf physiological and metabolic parameters in oak. Correlations between SWC and (**a**) stomatal conductance (SC), **b**) pre-dawn leaf water potential (Ψ_{PD}), (**c**) leaf osmotic potential (π), and (**d**) carbon isotope ratio ($\delta^{13}C$) in the soluble fraction were derived from the data collected for 72 *Q. robur* (green) and 10 *Q. petraea* (yellow) trees from sets 1 and 2 during the first 11 days after the water withdrawal (before any tree included in the experiment was rewatered), under extreme drought (SWC < 12%), and after four days of recovery. The correlation between SWC and SC, π, and $\delta^{13}C$ is best described by linear regression. The correlation between SWC and Ψ_{PD} is best described by a bilinear regression fit for SWC < 18% and SWC > 15%, respectively. The 15% and 16.6% SWC threshold points (the intersection of the lines) for *Q. robur* and *Q. petraea*, respectively, were calculated using the equations of the two regression lines. The SWC threshold point is the level of SWC at which a steep decline in Ψ_{PD} occurred in most of the oak trees included in the experiment. To account for the variation between plants, we collected the extreme drought samples at SWC slightly below this threshold (SWC ≤ 12%).

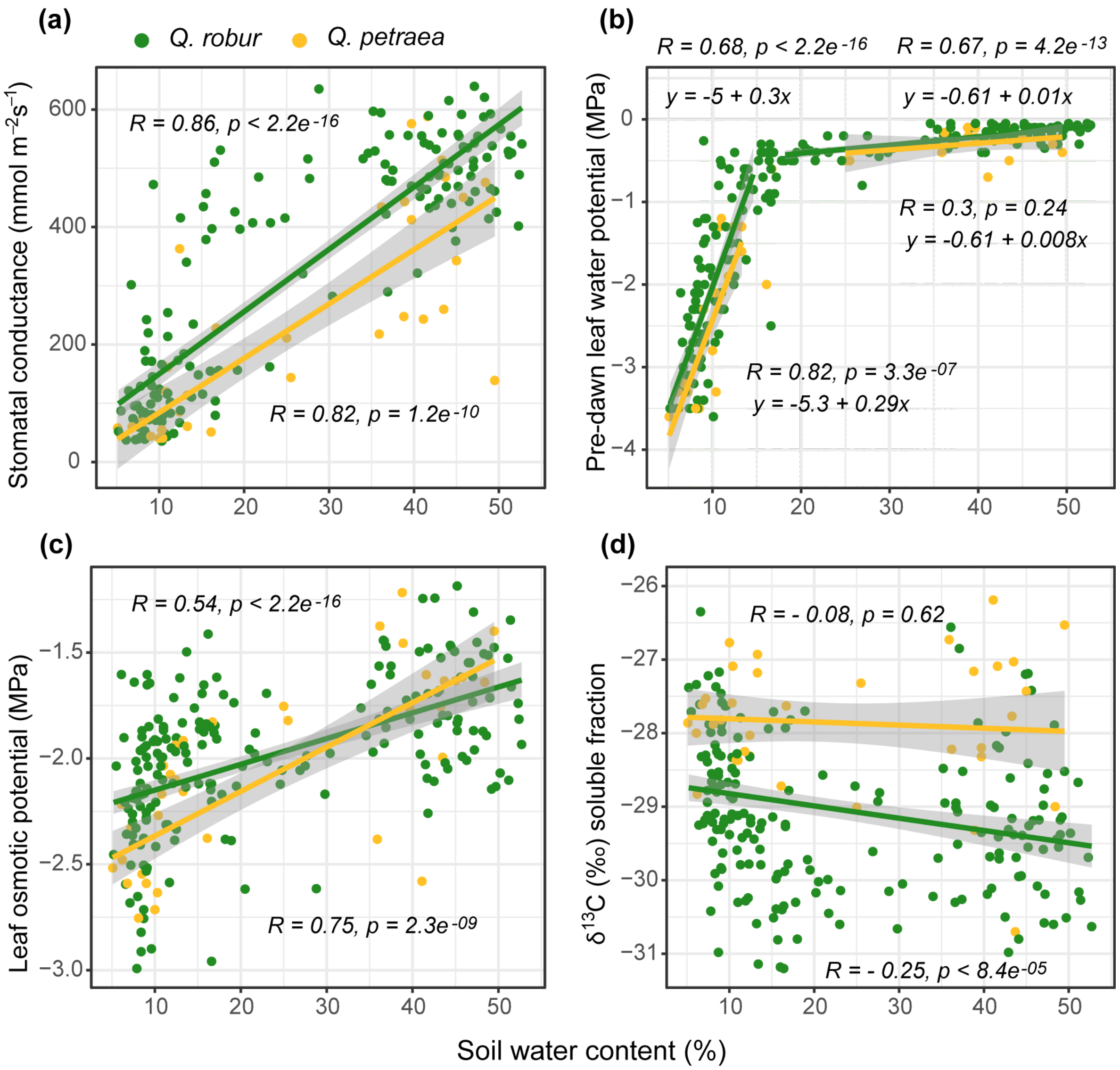

Figure S3. Drought response in *Quercus robur* and *Q. petraea* trees from population P2 (Northeast German lowlands). **(a, b)** Carbon isotope ratio ($\delta^{13}C$) in soluble and solid leaf tissue fractions, **(c)** leaf osmotic potential (π), **(d)** carbon (C) and **(e)** nitrogen (N) content, and **(f)** carbon-to-nitrogen ratio (C/N) in leaves are shown for 25 *Q. robur* and 58 *Q. petraea* trees from population P2 under normal irrigation and extreme drought (SWC < 12%) conditions. Due to the low N content in the soluble fraction, N content and C/N ratio are shown only in the solid fraction. *Wilcoxon test P-value < 0.5. The thick line in each box shows the median. The boxplot method is as in Fig. S3.

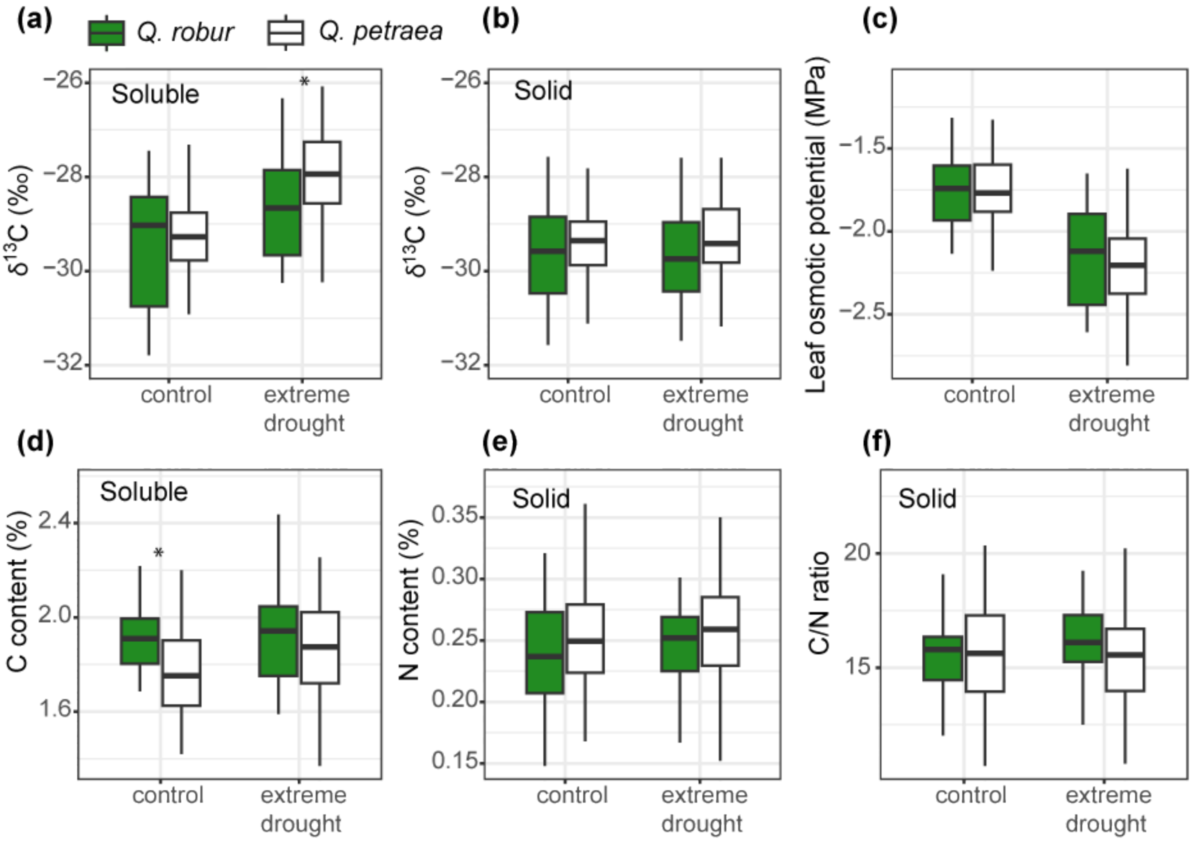

Figure S4. Comparison of the N and C contents in leaves of *Quercus robur* and *Q. petraea* during the drought stress experiment. Dynamics of changes in (**a, b**) C and (**c, d**) N content and (**e, f**) C/N ratio are shown for the soluble and solid fractions of leaf tissue in *Q. robur* and *Q. pertraea* during the first 11 days after water withdrawal (before re-watering of any tree), under extreme drought, and after four days of recovery. The day 2 corresponds to control. The onset of "extreme drought" corresponds to the day when SWC fell below 12%; it varied from 11 to 21 days after water withdrawal depending on a plant. Plots were constructed based on data for 72 *Q. robur* and 10 *Q. petraea* trees from sets 1 and 2. Stars indicate significant differences between species (Wilcoxon test P-value * < 0.05; ** < 0.01). The lower and upper limits of the box (hinges) correspond to the first and third quartiles, respectively. The upper whiskers extend to the maximum value but no further than 1.5 of the inter-quartile range (IQR) from the hinge. The lower whiskers extend to the minimum value, but no further than 1.5 IQR from the hinge.

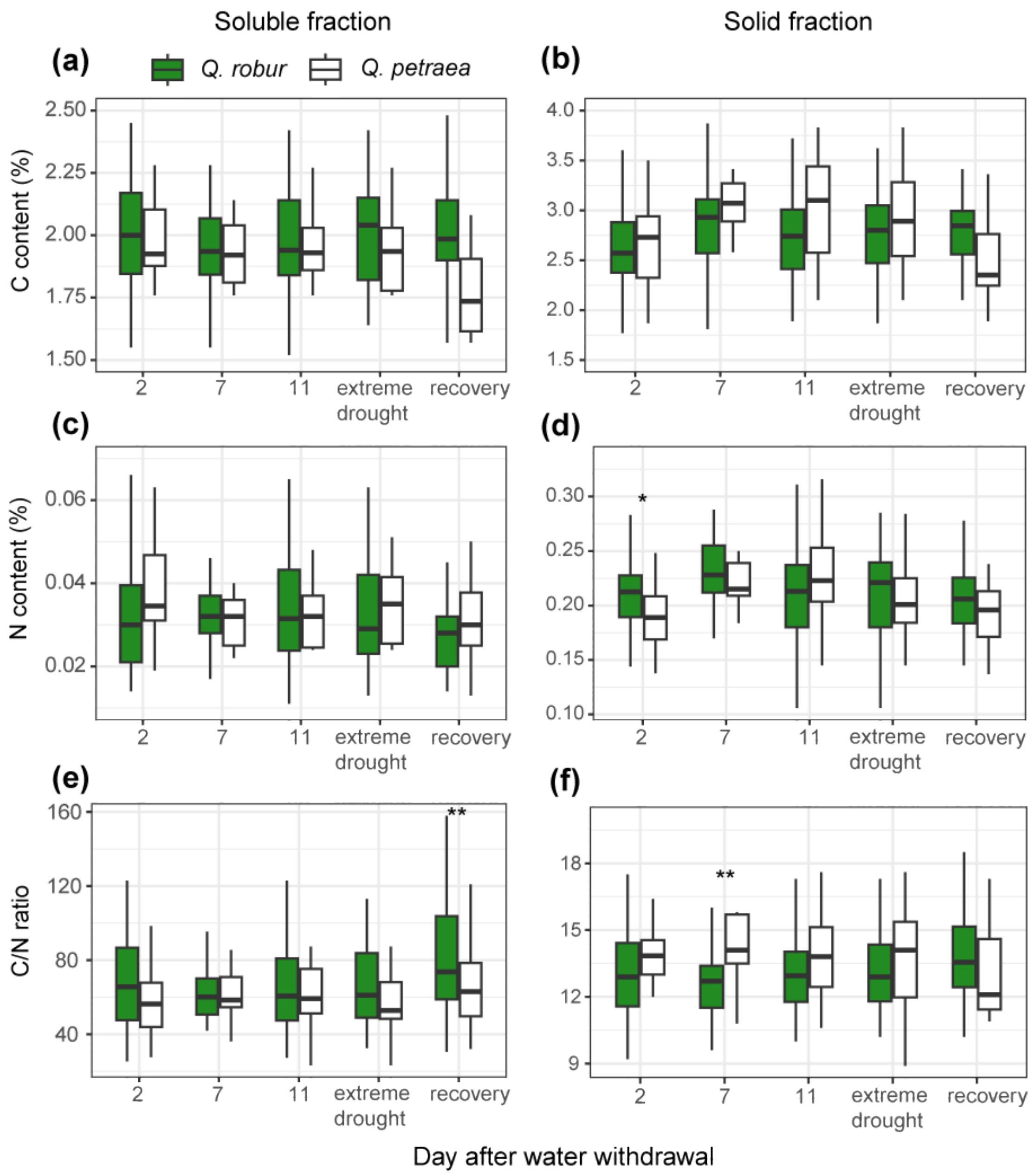

Figure S5. Correlations between the drought response traits in *Quercus robur* from seven German populations. Correlations between traits were inferred from the data collected for the 519 *Q. robur* trees under normal irrigation and extreme drought conditions. Carbon isotope ratio ($\delta^{13}C$), N and C content, and C/N ratio are given for the soluble leaf fraction. Circle size and color intensity correspond to Pearson's correlation values as shown in the color scale. Only significant correlations ($P_{adj} < 0.05$) are shown.

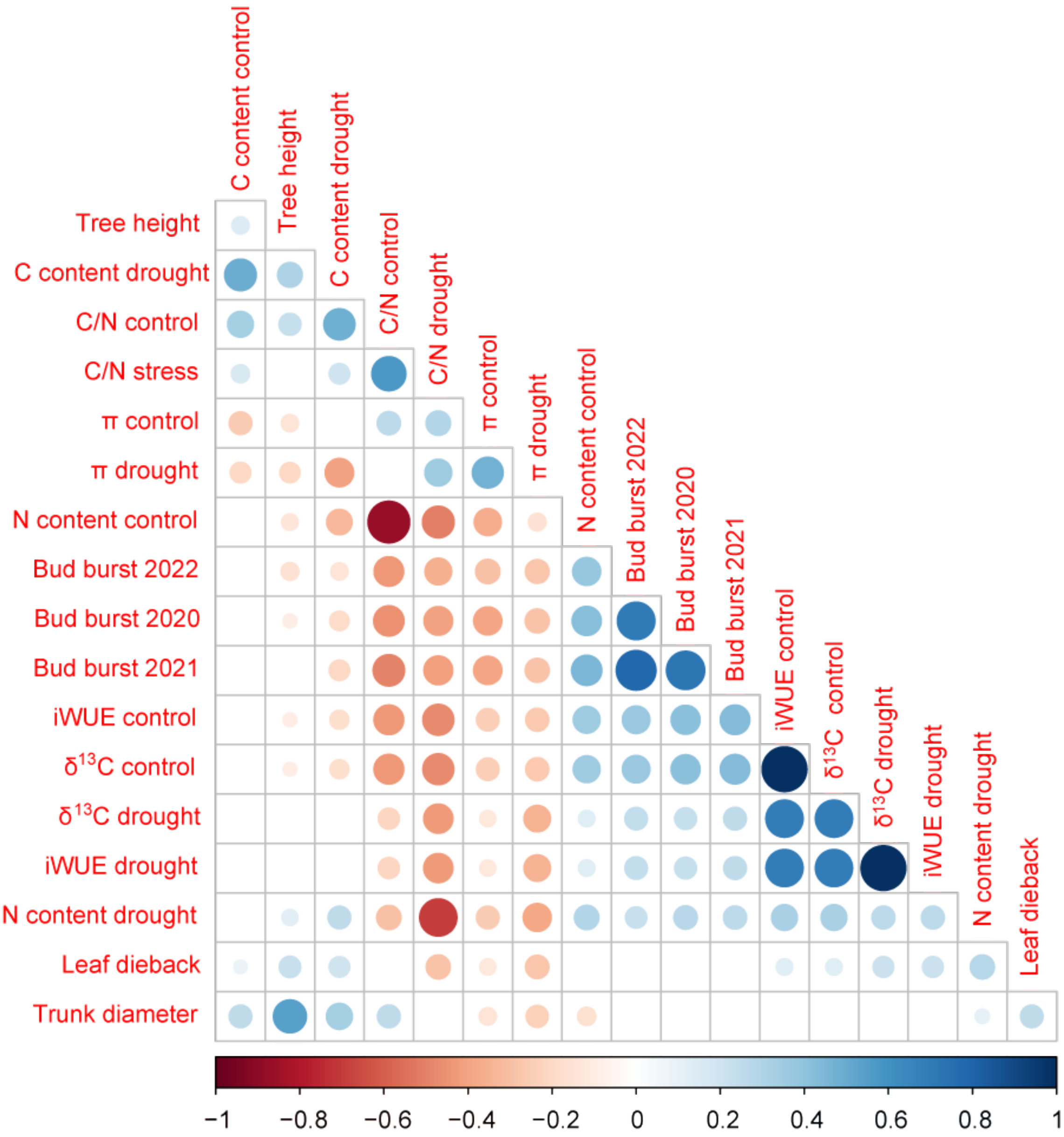

Figure S6. Effects of location at the experimental site and bud burst time on the distribution of carbon isotope ratio (δ^{13}) in oak trees from seven German populations. (**a-c**) Distribution of δ^{13} across the common garden experimental area is shown under (**a**) normal irrigation and (**b**) extreme drought conditions and (**c**) δ^{13}C plasticity in the soluble fraction of leaves in *Quercus robur* and *Q. petraea*. The outlined cells correspond to the nine-tree blocks (see Fig. S2b). The white line separates the groups of early and late bud burst (BB) trees. Trees were divided into the two groups based on the BB observations in the years 2020 and 2021 (Table S2). The average two-year difference in BB between the two groups was one week. (**d-f**) δ^{13}C under (**d**) normal irrigation and (**e**) drought conditions and (**f**) δ^{13}C plasticity in the early and late BB *Q. robur* trees from different populations. The number of early and late BB *Q. robur* trees in each population is shown above the x-axis in (e). The order of populations corresponds to the moisture deficit (MD; low to high) in the regions of population origin. Stars indicate significant differences in δ^{13}C and δ^{13}C plasticity between the early and late BB trees: Wilcoxon test P-value * < 0.05, ** < 0.01, *** < 0.001. The statistical method for the boxplot is as in Fig. S2. This figure shows strong BB time effect on δ^{13}C and δ^{13}C plasticity distributions: δ^{13}C in early BB trees is higher compared to late BB trees under both normal irrigation and extreme drought conditions. However, the amplitude of the drought-induced adjustments (i.e., δ^{13}C plasticity) is higher in the late BB trees. No location effect is observed on the distribution of δ^{13}C and δ^{13}C plasticity. The air temperature distribution across the experimental area is shown in Fig. S1.

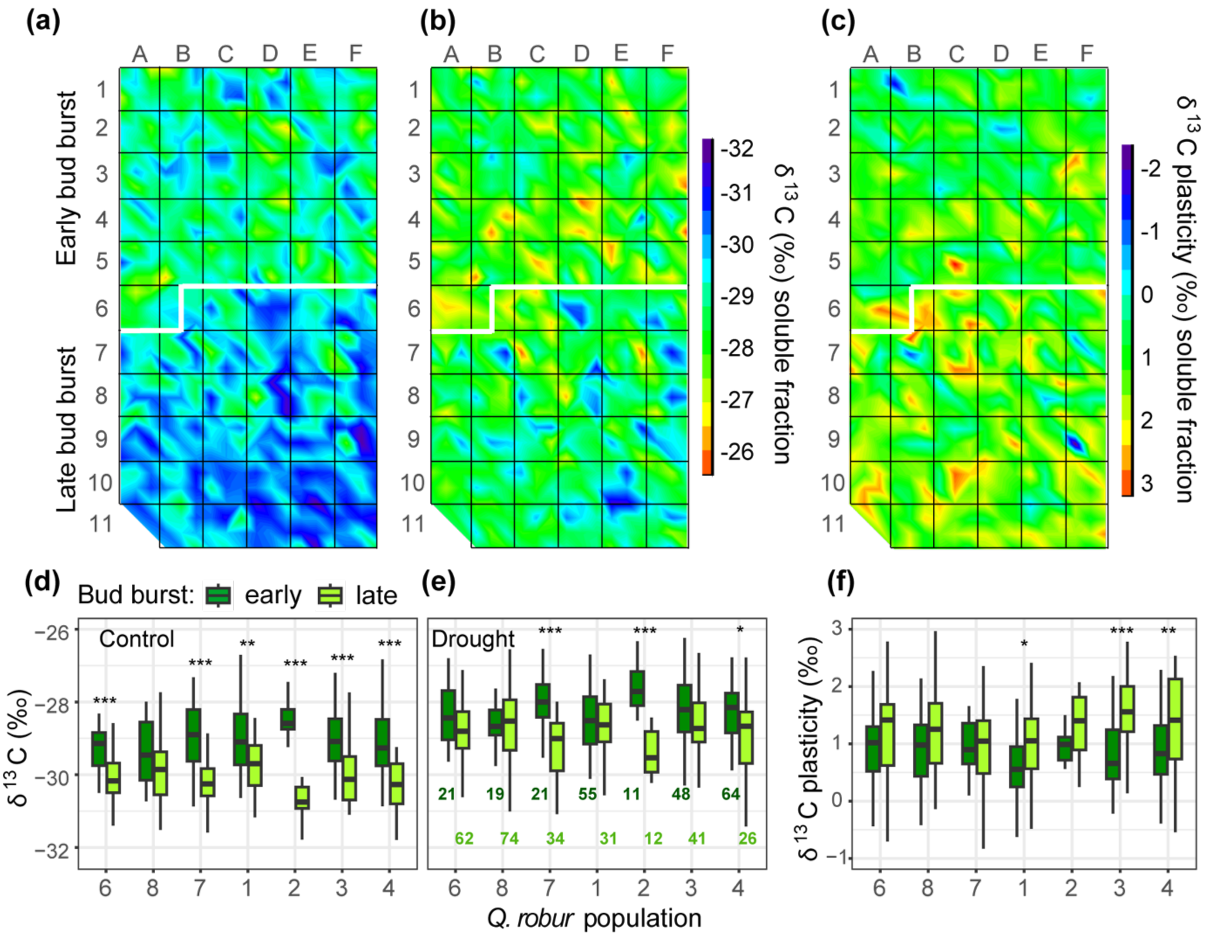

Figure S7. Effects of location at the experimental site and bud burst time on leaf osmotic potential in oak trees from seven German populations. The heat maps show the distribution across the common garden trial area of (**a, b**) leaf osmotic potential (π) under normal irrigation and extreme drought conditions and (**c**) π plasticity in 519 *Quercus robur* and 60 *Q. petraea* trees. Outlined cells correspond to the blocks of nine trees (see Fig. S1b). The white lines separate the groups of early and late bud burst (BB) trees. Trees were divided into the two groups based on the BB observations in the years 2020 and 2021 (Table S2). The two-year average difference in BB time between the two groups is one week. (**d-f**) π under normal irrigation and extreme drought conditions and (**f**) π plasticity in the early and late BB *Q. robur* trees from different populations. The number of early and late BB *Q. robur* trees in each population is shown above the x-axis in (e). The order of populations corresponds to the soil moisture deficit (MD; low to high) in the regions of population origin. Stars indicate significant differences in π between the early and late bud burst trees: Wilcoxon test P-value * < 0.05, ** < 0.01, *** < 0.001. The statistical method for the boxplot is as in Fig. S2. This figure shows a strong BB time effect on π: π in the early BB trees is lower compared to late BB trees under both normal irrigation and extreme drought conditions. The amplitude of the drought-induced osmotic adjustments (i.e., π plasticity) does not differ between the two groups. No location effect on the distribution of π and π plasticity was observed. The temperature distribution across the experimental area is shown in Fig. S1.

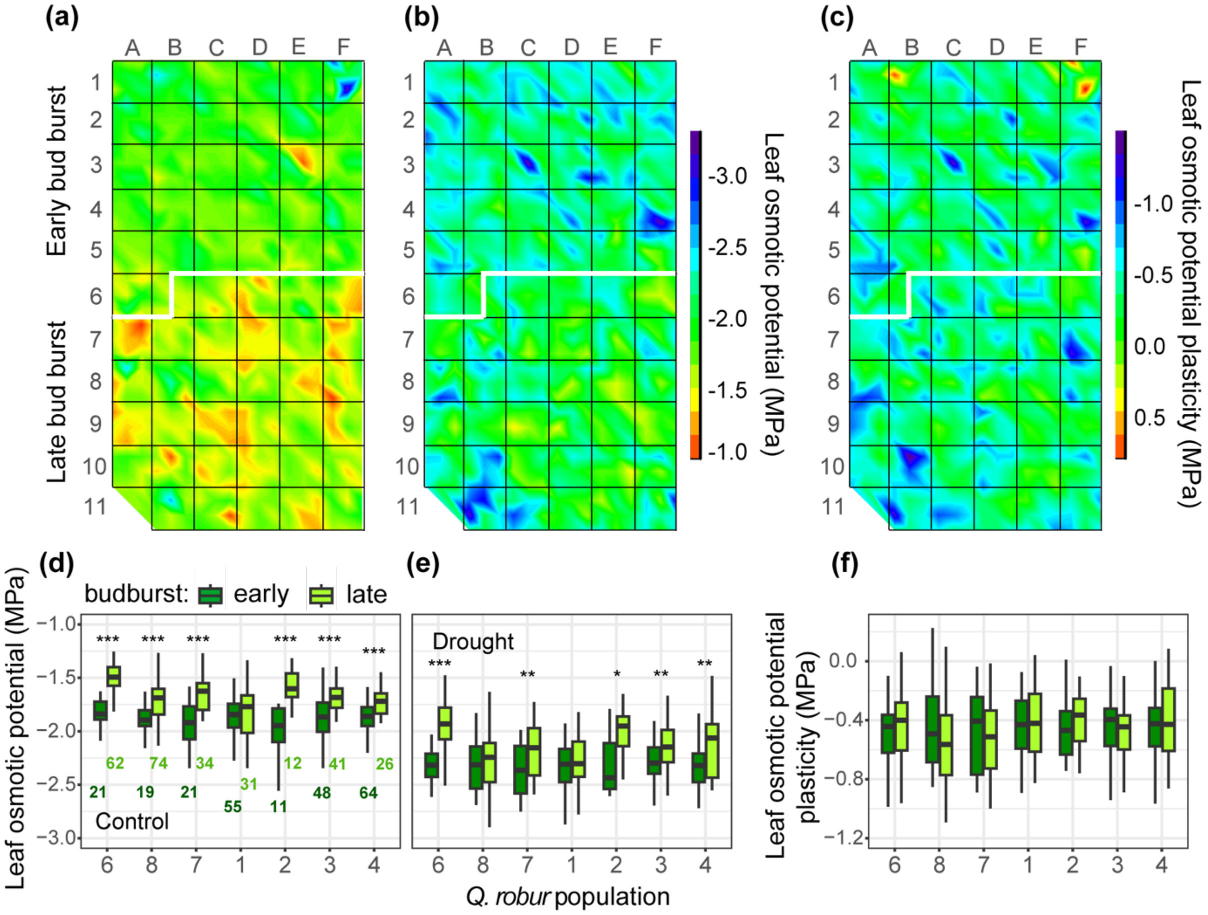

Figure S8. Air temperature at the common garden site at Großhansdorf, Germany during leaf development in oak trees in 2022. (**a**) Leaf development in the oak trees in 2022. The dashed curve shows percent of trees that have reached the bud swelling stage (stage 1; Bertic *et al.*, 2021). The dotted curve shows percent of trees at the leaf unfolding stage or above. The solid black curve shows percent of trees with fully expanded leaves (stage 5). (**b**) Mean and minimum (gray and blue curves, respectively) daily temperatures at the common garden site during leaf development in oak trees in 2022. The vertical green line corresponds to the date when leaf unfolding was completed in the early bud burst (BB) trees. This figure shows several episodes of chilling temperatures ($0 < T \leq 5°C$) after leaf unfolding in both early and late BB trees. This allows to exclude priming by low temperature in early BB trees only as a possible reason for the differences in carbon isotope ratio and leaf osmotic potential observed between the two groups.

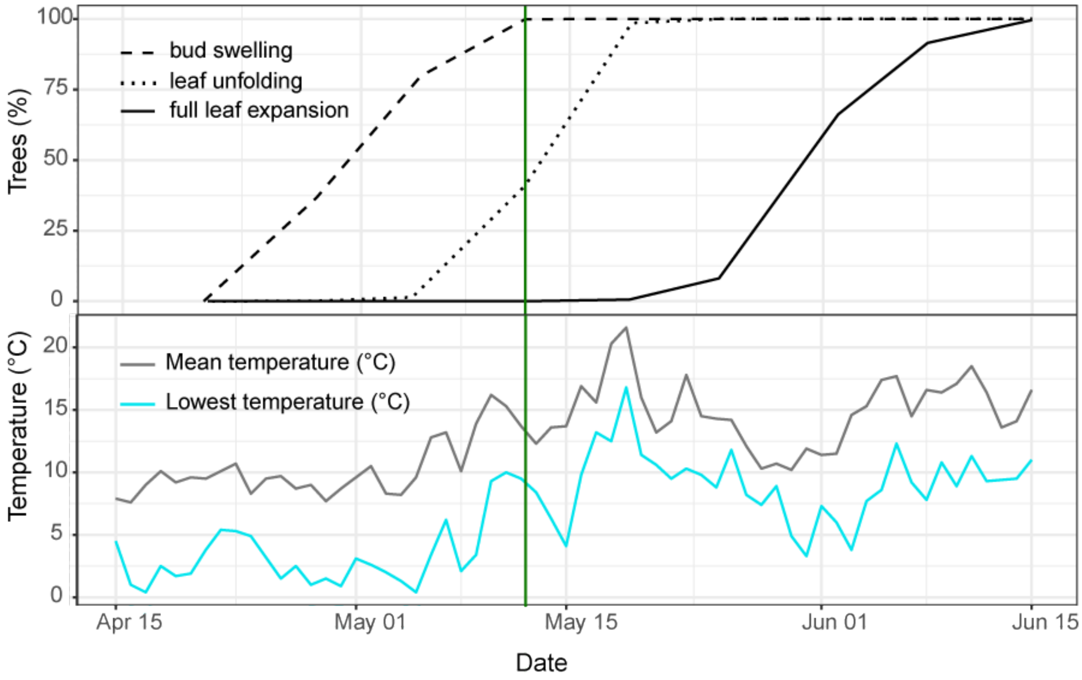

Figure S9. Correlation between carbon isotope ratio ($\delta^{13}C$) in the soluble fraction of *Quercus robur* leaves and climatic and soil parameters. Correlation is shown between the population median $\delta^{13}C$ in the soluble fraction of leaves collected under control (gray dots) and extreme drought (red triangles) conditions and the mean (**a**) annual precipitation, (**b**) precipitation of the vegetation period, (**c**) precipitation of the driest month (i.e., April), (**d**) aridity index (Ia), (**e**) soil water holding capacity (FC), and (**f**) soil air volume (AC) of the vegetation period in the regions of origin of the seven *Q. robur* populations included in this study. These plots were constructed based on the data for 519 *Q. robur* trees.

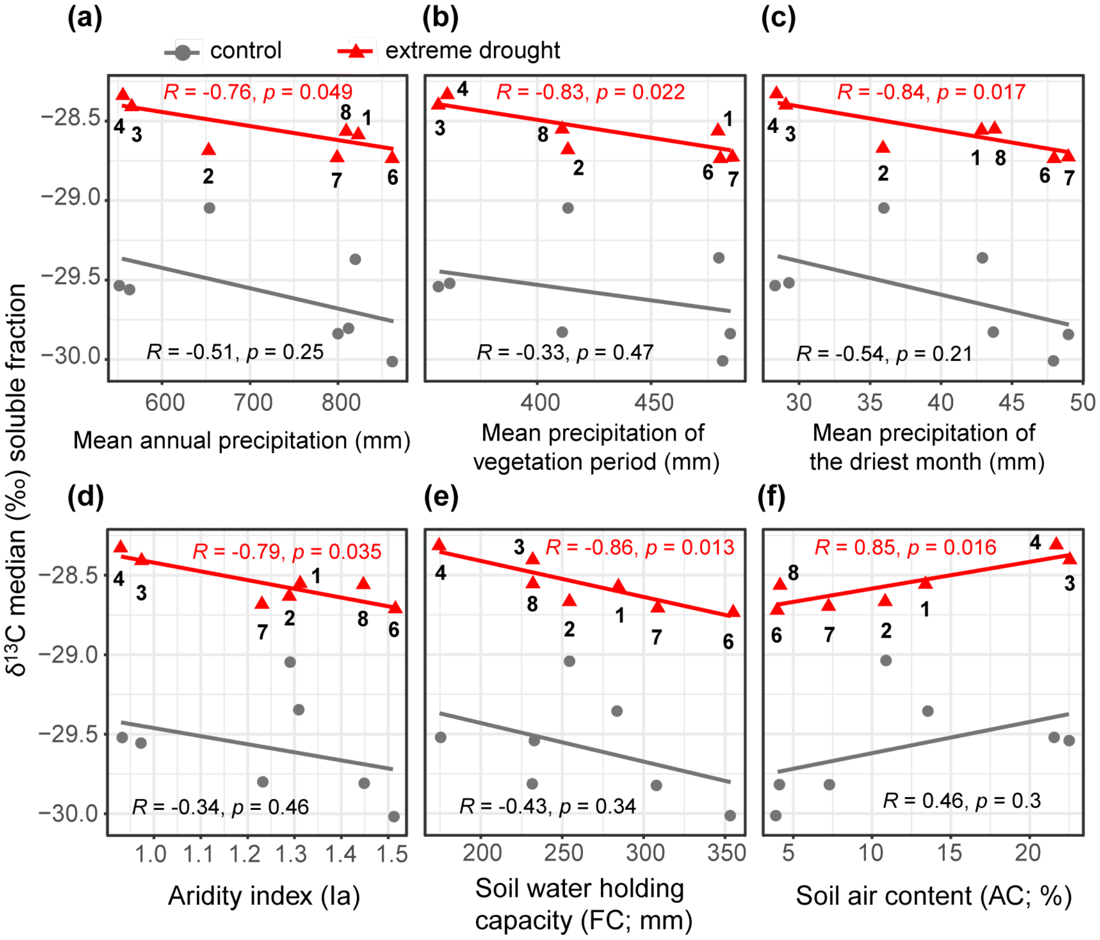

Figure S10. Correlation between climate and soil variables in the regions of oak population origin in Germany. Circle size and color intensity correspond to the Pearson correlation between two climate/ soil variables at the origin regions of seven oak populations included in this study. Only significant correlations ($P_{adj} < 0.05$) are shown. Abbreviations are explained in Table S1.

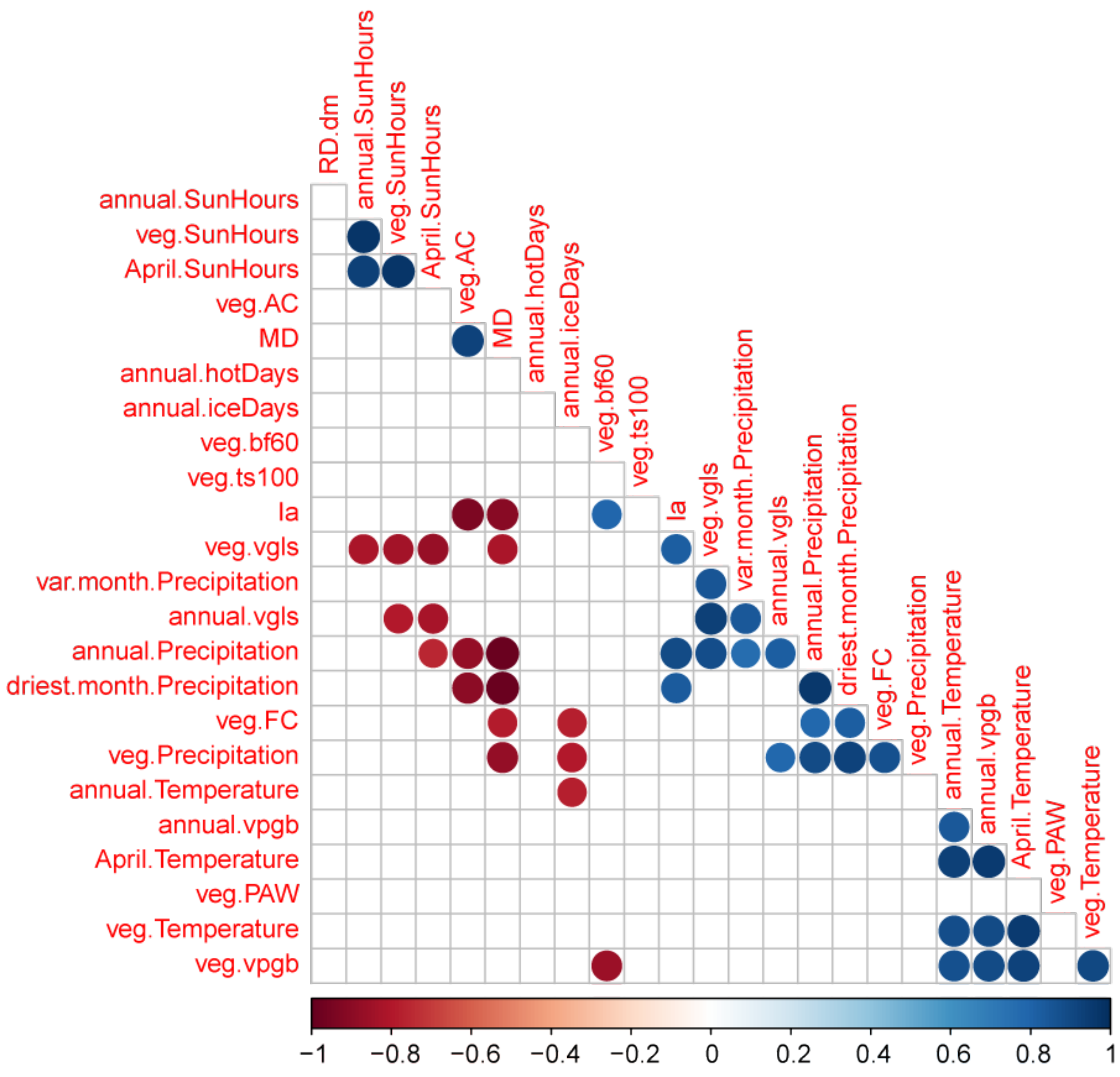

Figure S11. Climatic clines in the distribution of carbon isotope ratios in *Quercus robur* trees with early and late bud burst. (**a**) Correlation between the median carbon isotope ratio ($\delta^{13}C$) in trees with early (dark green circles) and late (light green triangles) bud burst (BB) under normal irrigation and extreme drought conditions and mean annual number of hot days (T > 25°C) in the population origin regions. (**b**) Correlation between mean number of hours of sunshine in April and moisture deficit (MD) in the origin regions of seven *Q. robur* population. (**c**) Lack of correlation between mean annual number of hot days and MD in the *Q. robur* population origin regions. This figure shows that $\delta^{13}C$ forms opposite clines in early and late BB trees when populations are distributed by the annual number of hot days. The mean number of sunshine hours correlates with MD in the *Q. robur* population origin regions, but the mean annual number of hot days does not. The correlation between $\delta^{13}C$ and the mean number of sunshine hours in April is shown in Figure 4b.

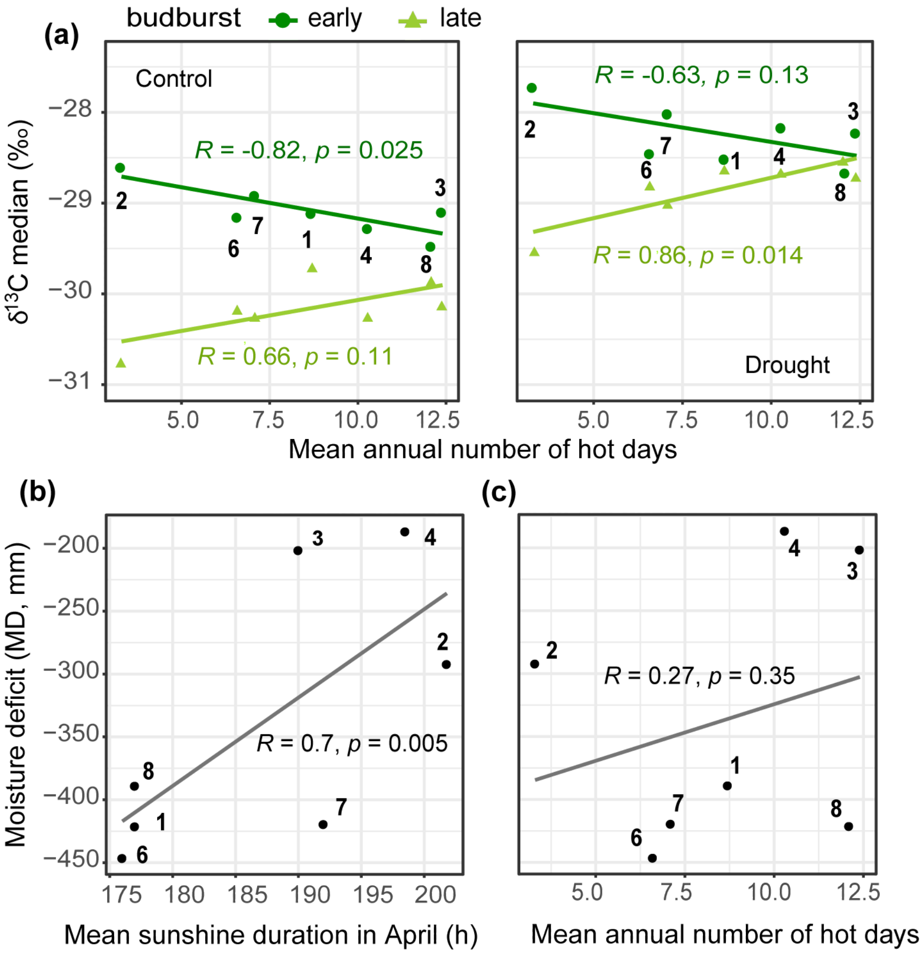

Figure S12. Correlation between intrinsic water use efficiency (iWUE) values in soluble and solid fractions of the oak leaf tissue. Correlation between iWUE in soluble and solid leaf tissue fractions were inferred from carbon isotope ratio ($\delta^{13}C$) measured in 519 *Quercus robur* and 60 *Q. petraea* common garden trees under normal irrigation and extreme drought conditions. Point colors correspond to populations. Population IDs are as in Fig. S1. Regression line is in red. The gray shading represents the 95% confidence interval of the regression line.

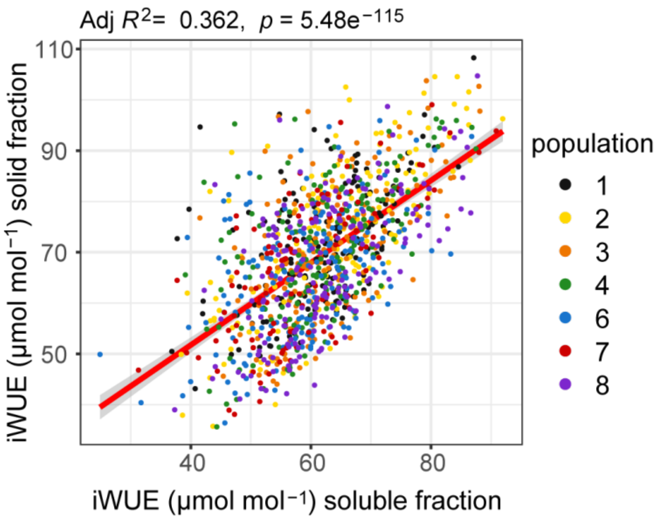

Figure S13. Correlation between intrinsic water use efficiency (iWUE) in oak leaves under normal irrigation and extreme drought conditions. iWUE was calculated based on carbon isotope ratio ($\delta^{13}C$) in **(a)** soluble and **(b)** solid fractions of oak leaf tissue. Data includes measurements for 519 *Quercus robur* and 60 *Q. petraea* trees included in the common garden drought stress experiment conducted under normal irrigation and extreme drought conditions. Point colors correspond to populations. Population IDs are as in Fig. S1. Regression lines are in red. The gray shading represents the 95% confidence interval of the regression line.

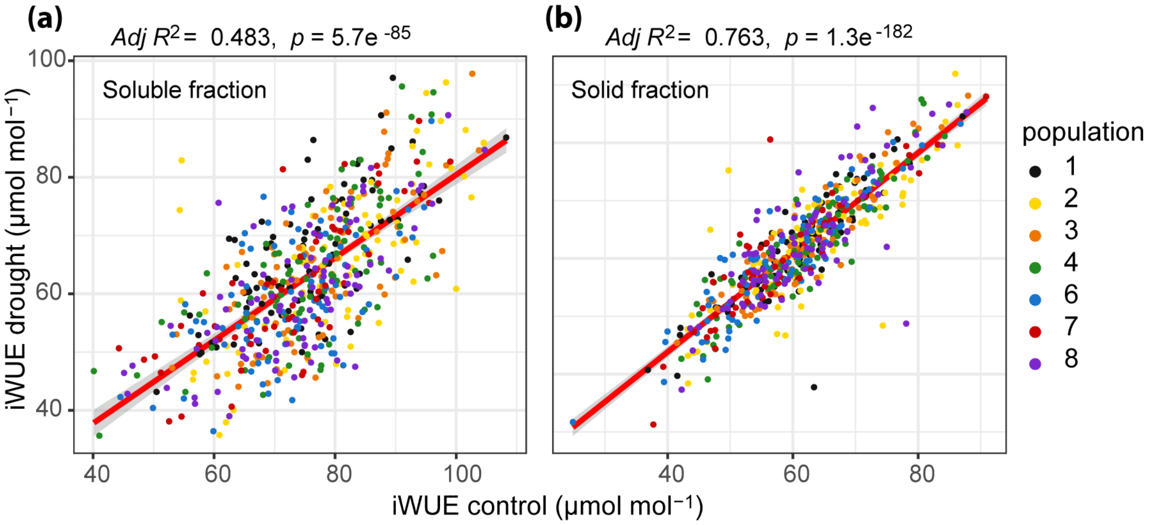

Figure S14. Trait variation between selected *Quercus robur* genotypes with high and low intrinsic water use efficiency (iWUE). Comparison of (**a**) nitrogen (N) content, (**b**) carbon (C) content, (**c**) carbon-to-nitrogen ratio (C/N) values in the soluble and solid leaf tissue fractions, and (**d**) leaf osmotic potential (π) under normal irrigation and extreme drought conditions and (**e**) carbon isotope ratio ($\delta^{13}C$) and π plasticity (i.e., drought-induced change) between the *Q. robur* genotypes characterized by high and low iWUE (T [green] and S [brown], respectively). Stars indicate significant differences between T and S: Wilcoxon test P-value * < 0.05, ** < 0.01, *** < 0.001. The statistical method of the box-plots is as in Fig. S2. (**f**) Drought-induced leaf dieback in T and S *Q. robur* genotypes from seven German populations. Dark brown indicates the number of T and S trees with dieback > 5% of canopy leaves; light brown indicates the number of trees with dieback ≤ 5% of canopy leaves.

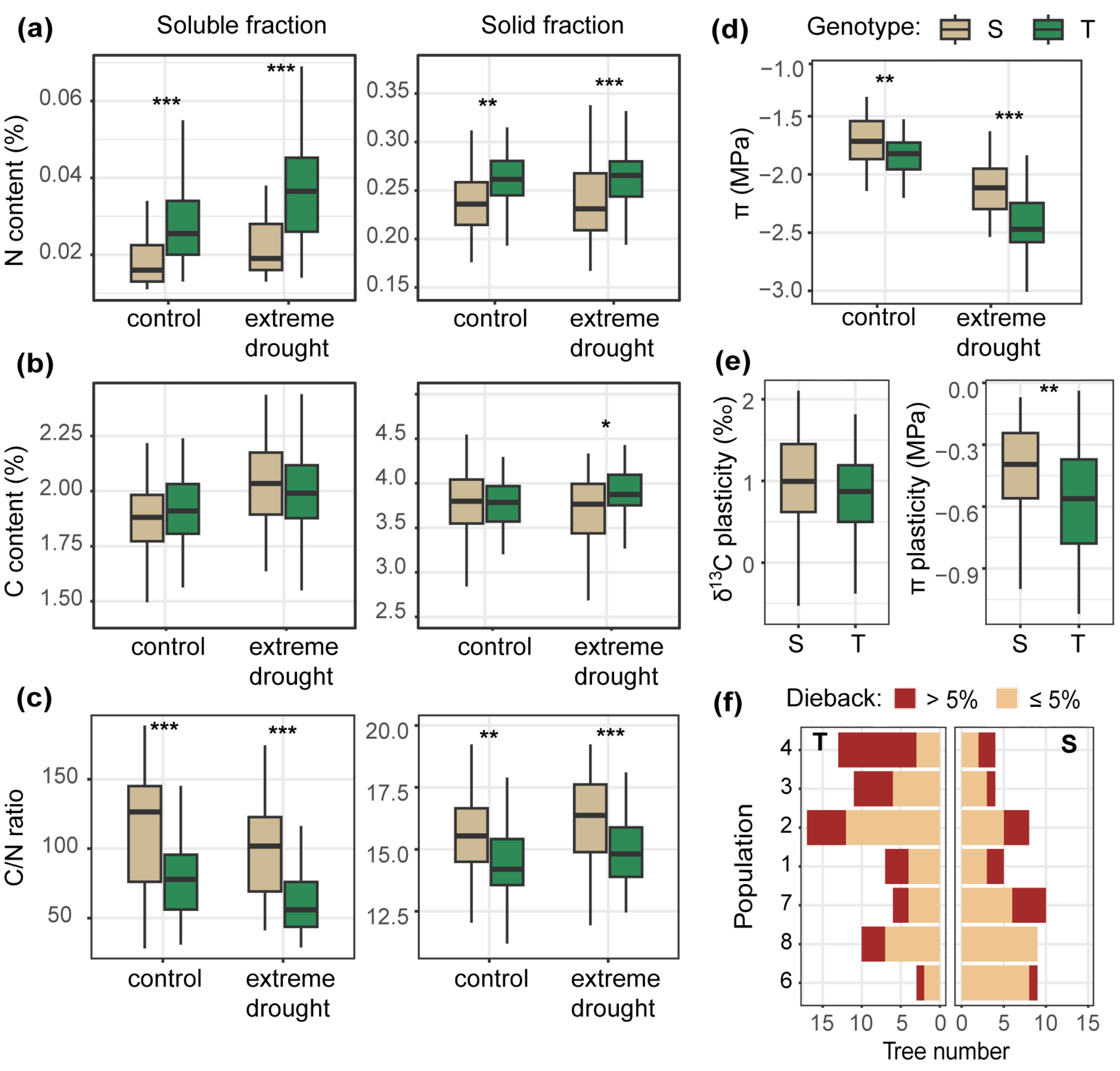

Figure S15. Correlation between the concentration of abscisic acid (ABA) and intrinsic water efficiency (iWUE) and leaf osmotic potential (π) in selected drought-tolerant and susceptible oak genotypes. Correlations of ABA concentration with (**a**) iWUE and (**b**) π are shown in leaves of T and S oak genotypes under normal irrigation and extreme drought conditions.

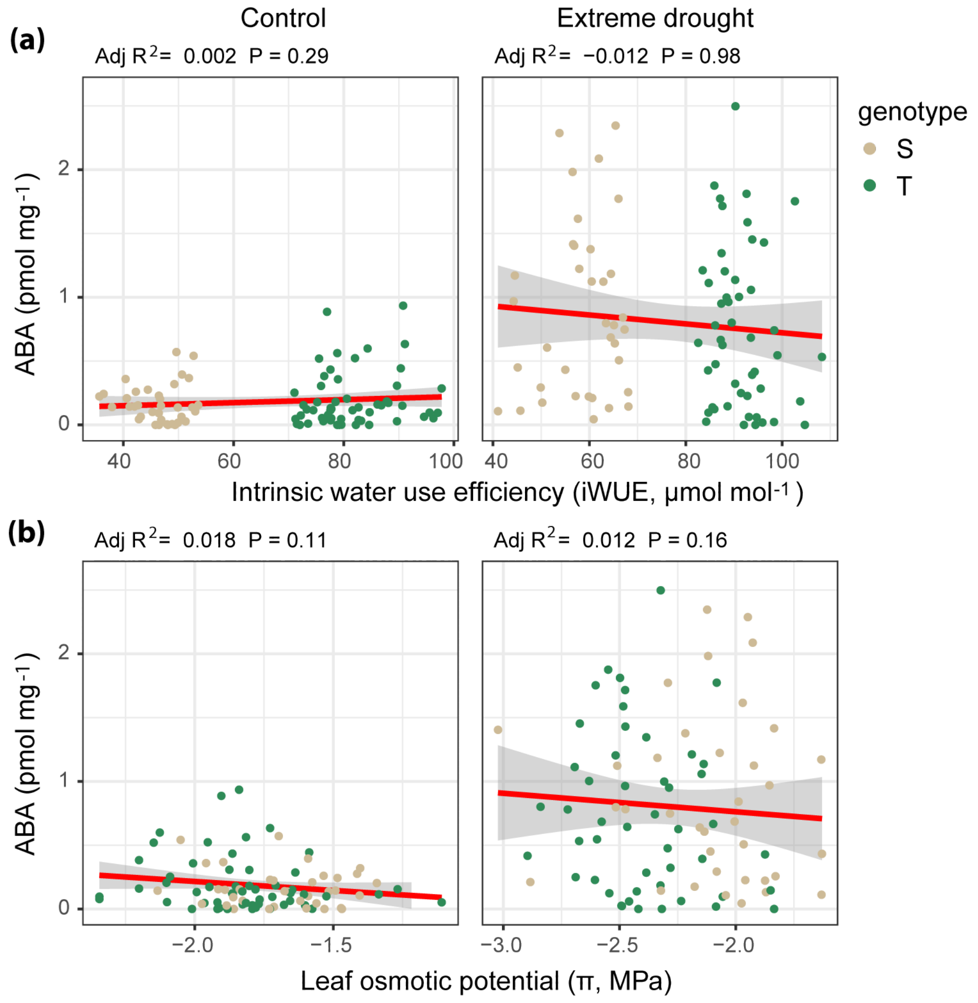

Figure S16. Trait variation in the *Quercus robur* cpDNA haplotypes in Germany. (**a**, **b**) Variation in carbon isotope ratio ($\delta^{13}C$) between the *Q. robur* cpDNA haplotypes under (**a**) normal irrigation and (**b**) extreme drought conditions. Plots are constructed based on $\delta^{13}C$ in the soluble fraction of leaves sampled from 519 *Q. robur* trees. Population IDs are as in Fig. 1. Statistical method for boxplots is as in Fig. S2. Frequency of trees with (**c**) early (dark green) and late (light green) time of bud burst (BB) and (**d**) high (dark brown) and low (light brown) drought-induced leaf dieback in different *Q. robur* cpDNA haplotypes in Germany. The numbers at the top of each of segment indicate the percentage of trees in each group from the total of 519 *Q. robur* trees included in this study. CpDNA haplotype IDs are as in Petit *et al.* (2022): haplotype 1 originating from the Italian peninsula, haplotypes 10, 11, and 12 from the Iberian Peninsula, haplotypes 4, 5, and 7, from the Balkans. Our analysis does not distinguish between the three Iberian haplotypes 10, 11, and 12. This figure shows no significant association between the *Q. robur* cpDNA haplotypes and $\delta^{13}C$, BB time, and intensity of the drought-induced leaf dieback. No significant difference between haplotypes was also observed for other traits, including N and C content, C/N ratio in oak leaves, and leaf osmotic potential (data are not shown).

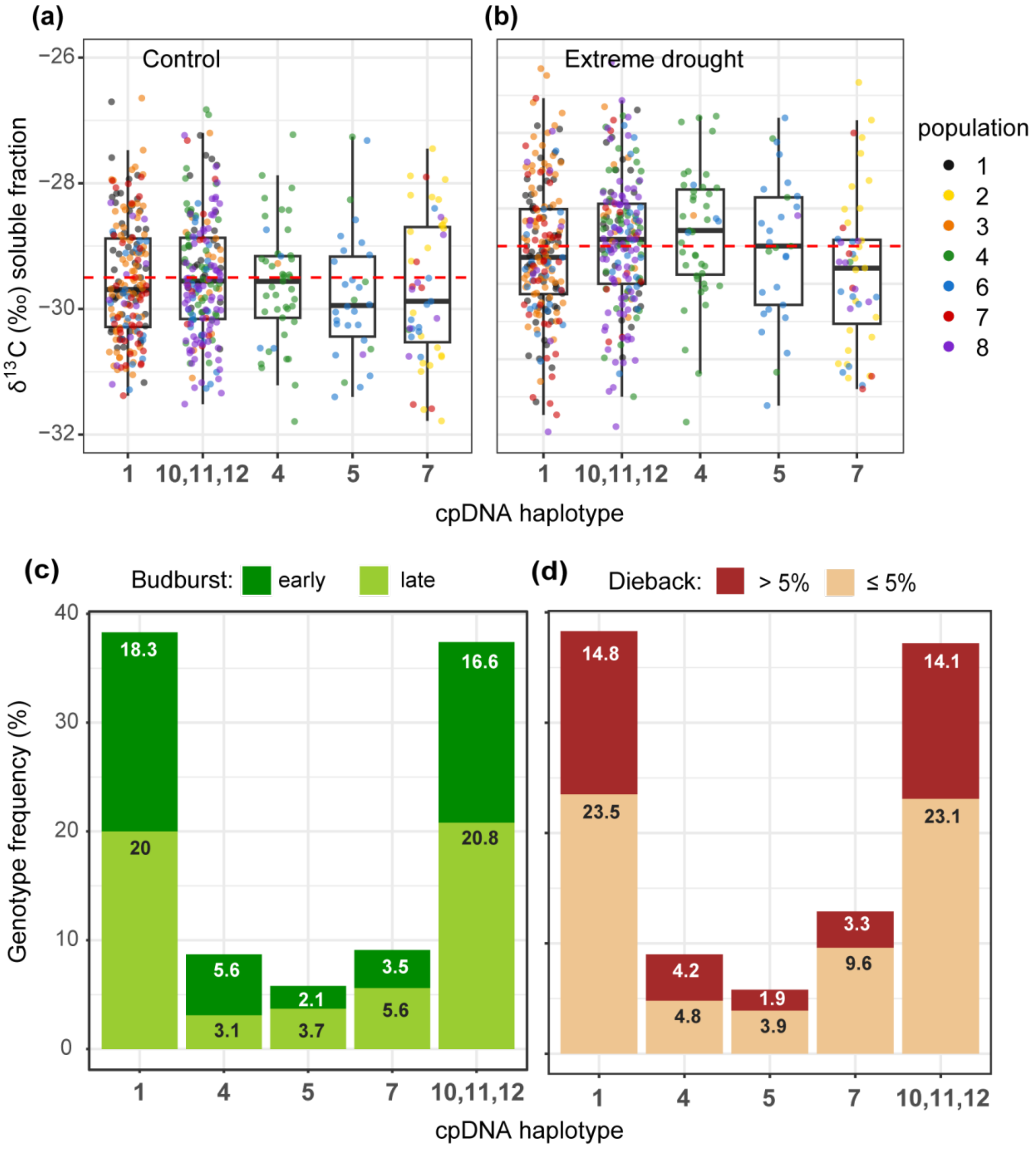

References

Bertic M., H. Schroeder, B . Kersten, M. Fladung, F. Orgel, F. Buegger, et al. 2021. “European oak chemical diversity - from forest stands to herbivore resistance”. *New Phytologist 232*(2): 818-834.

Petit R.J., S. Brewer, S. Bordács, K. Burg, R. Cheddadi, E. Coart, J. Cottrell J, et al. 2002. “Identification of refugia and post-glacial colonisation routes of European white oaks based on chloroplast DNA and fossil pollen evidence”. *Forest Ecology and Management 156*(1-3): 49-74.

Zhou R., T.M. Squires, S.J. Ambrose, S.R. Abrams, A.R.S. Ross, and A.J. Cutler. 2023. “Rapid extraction of abscisic acid and its metabolites for liquid chromatography–tandem mass spectrometry”. *Journal of Chromatography A 1010*: 75-85.
